# Association Between Regular Use of Gastric Acid Suppressants and Subsequent Risk of Cholelithiasis: A Prospective Cohort Study of 0.47 Million Participants

**DOI:** 10.3389/fphar.2021.813587

**Published:** 2022-01-28

**Authors:** Man Yang, Bin Xia, Yawen Lu, Qiangsheng He, Yanyan Lin, Ping Yue, Bing Bai, Chunlu Dong, Wenbo Meng, Jian Qi, Jinqiu Yuan

**Affiliations:** ^1^ The First Clinical Medical School of Lanzhou University, Lanzhou, China; ^2^ The Department of General Surgery, The First Hospital of Lanzhou University, Lanzhou, China; ^3^ Guangdong Provincial Key Laboratory of Gastrointestinal Cancers, Center for Digestive Disease, The Seventh Affiliated Hospital, Sun Yat-sen University, Shenzhen, China; ^4^ Clinical Research Center, The Seventh Affiliated Hospital, Sun Yat-sen University, Shenzhen, China; ^5^ Big Data Centre, The Seventh Affiliated Hospital, Sun Yat-sen University, Shenzhen, China

**Keywords:** proton pump inhibitor, H2-receptor antagonists, choletithiasis, cohort study, risk factor

## Abstract

**Background:** Gastric acid suppressants have a major impact on gut microbiome which in turn, may increase the risk of cholelithiasis, but epidemiological evidence remains unclear. We undertook this research to evaluate the association between regular use of proton pump inhibitors (PPIs) and H2-receptor antagonists (H2RAs) with risk of cholelithiasis.

**Methods:** Prospective cohort study included 477,293 UK residents aged 37–73 years from the UK Biobank. We included the participants reported PPI or H2RA use, and were free of cholelithiasis or cancer. We evaluated hazard ratios (HRs) of regular use of PPIs or H2RAs and risk of cholelithiasis adjusting for demographic factors, lifestyle habits, the presence of comorbidities, use of other medications, and clinical indications.

**Results:** We identified 12,870 cases of cholelithiasis over a median follow-up of 8.1 years. Regular use of PPIs (HR 1.22 95% CI 1.16–1.29) or H2RAs (HR 1.16, 95% CI 1.05–1.28) was associated with an increased risk of cholelithiasis after confounding adjustment. There were no major differences among individual PPIs/H2RAs. The absolute risk of PPI-associated cholelithiasis was increased with the baseline predicted risk evaluated by known environmental and genetic risk factors (Risk differences in the lowest vs. the highest quartile: 1.37 vs. 4.29 per 1,000 person-years).

**Conclusion:** Regular use of PPIs and H2RAs was associated with increased risk of cholelithiasis. Future prospective studies are required to confirm whether the observed associations are casual.

## Introduction

Cholelithiasis, or gallstone, is one of the most common gastrointestinal diseases. The prevalence of gallstone disease ranged from 5.9 – 21.9% in Europe (Aerts and Penninckx, 2003). In the USA, over 20.5 million people (15% of the population) were estimated to have gallstones, with an annual cost for the treatment and prevention of this disease over 62 billion (Everhart and Ruhl, 2009). A number of factors, such as family history, female sex, and low-grade physical activity, have been shown to be associated with increased risk of cholelithiasis (Pak and Lindseth, 2016). Recent research revealed that intestinal flora imbalance may affect bile acid metabolism which, in turn, promote the formation of gallstone (Wu et al., 2013; Molinero et al., 2019; Wang et al., 2020).

Gastric acid suppressants, including the proton pump inhibitors (PPIs) and H2-receptor antagonists (H2RAs), are commonly used for the treatment of gastroesophageal reflux disease (GERD), peptic ulcer disease, and for the prevention of nonsteroidal anti-inflammatory drug-(NSAID) induced gastrointestinal injury (Freedberg et al., 2017). Owning to the wide indications and large population of patient, acid suppressants are now one of the most commonly used medicines worldwide. Acid suppressants have a good short-term safety profile. However, cumulating studies showed that the long-term use of these medicines, particularly PPIs, were associated with various adverse health effects, such as fracture, chronic kidney disease, pneumonia, type 2 diabetes, and rheumatoid arthritis (Freedberg et al., 2017; He et al., 2020; Yuan et al., 2020; Yuan J et al., 2021). Recent studies also showed that PPI use was associated with biliary tract diseases, including cholangitis (Min et al., 2019), cholecystitis (Chuang et al., 2019), and bile duct cancer (Xiong et al., 2020; Kamal et al., 2021). It has been showed that acid suppressants may reduce gallbladder motility and lead to a delayed gallbladder emptying (Cahan et al., 2006), which in turn facilitates the development of gallstones. In addition, long-term use of acid suppressants may promote ductal epithelial proliferation, micropapillary growth of biliary epithelium, focal bile duct stricture formation, and bile duct obstruction (Yang et al., 2020). These morphological changes further facilitate the formation of gallstones. However, epidemiological research directly evaluating acid suppressants and risk of cholelithiasis is still lacking.

Given the widespread use of acid suppressants and high prevalence of gallstone diseases, investigation of their association would have a major impact on clinical practices and public health. In the present study, we undertook a prospective analysis of a large ongoing cohort, the UK Biobank, to evaluate the use of PPIs and H2RAs and subsequent risk of cholelithiasis.

## Materials and Methods

### Study Design

This is a prospective cohort study with a median follow-up of 8.1 years and analysis of over 0.47 million participants in the UK Biobank. The UK Biobank recruited about 500,000 UK residents aged 37–73 years from 2006 – 2010. At baseline, all included participants complete a touch screen questionnaire and face-to-face interview. A range of physical measurements, including height, weight, body composition, and blood pressure were taken, and biological samples were collected. Follow-up assessments were conducted through linkages to routinely available national datasets. Detailed description of this cohort can be found elsewhere (Sudlow et al., 2015). The UK Biobank has been used to investigate the association between regular use of PPIs and risk of diabetes (He et al., 2020), inflammatory bowel disease (Xia et al., 2021), and mortality (He et al., 2021).

In the current analysis, the participants were eligible for inclusion if they: 1) reported personal use of PPIs or H2RAs; 2) were free of cholelithiasis, cholecystitis, cholangitis, and cholecystectomy. We also excluded the participants 1) with a diagnosis of live cancer or pancreatic cancer, or 2) subsequently withdrew during follow-up (Supplementary Figure S1). The research was done with the UK Biobank resource as a part of the approved research application (no. 51671 extended scope). The UK biobank has been approved by the North West Multi-center Research Ethics Committee, the England and Wales Patient Information Advisory Group, and the Scottish Community Health Index Advisory Group (REC ID: 16/NW/0274). The return of the questionnaires from participants was considered implied consent. This study was also approved by the Ethics Committee of The Seventh Affiliated Hospital, Sun Yat-sen University (KY-2021-080-01).

### Assessment of PPI and H2RA Use

Regular use of PPIs or H2RAs was firstly assessed by participants using a touchscreen questionnaire. The participants were asked “*Do you regularly take any prescription medications?*”. ‘Regularly’ was routinely defined as most days of the week for the last 4 weeks. If participant selected ‘Yes’ or ‘Unsure’, then they would be asked by a trained staff: “*In the touch screen you said you are taking regular prescription medications. Can you now tell me what these are?*” The recorded types of PPIs included omeprazole, esomeprazole, lansoprazole, pantoprazole and rabeprazole. The kinds of H2RAs included ranitidine, cimetidine, famotidine and nizatidine.

### Assessment of Main Outcomes

Participants were followed through linkage to the Health and Social Care Information Centre (in England and Wales) and the National Health Service Central Register (in Scotland). The study outcome was the development of cholelithiasis, which was defined according to the International Classification of Diseases (ICD)-10 codes (*K80.0: Calculus of gallbladder with acute cholecystitis, K80.1: calculus of gallbladder with other cholecystitis, K80.2: calculus of gallbladder without cholecystitis, K80.3:calculus of bile duct with cholangitis, K80.4:calculus of bile duct with cholecystitis, K80.5:calculus of bile duct without cholangitis or cholecystitis, K80.6:other cholelithiasis*).

### Assessment of Covariates

Sociodemographic factors (age, sex, and ethnicity), lifestyle habits (smoking, alcohol consumption, and dietary intake), multivitamin use, and intake of mineral supplements were self-reported. Height and weight were measured by trained research staff. Index of multiple deprivation was provided directly from the UK Biobank. Physical activity was assessed using the International Physical Activity Questionnaire-Short Form (IPAQ-SF). Comorbidities (GERD, esophagitis, esophageal stricture, gastric or duodenal ulcer, upper gastrointestinal tract bleeding, gastritis, hypertension, hypercholesterolemia, diabetes), medication usage (aspirin, non-aspirin NSAIDs, statin, angiotensin-converting enzyme inhibitors [ACEIs], angiotensin II receptor blockers [ARBs], and beta-blockers) were assessed based on self-reported medical history and verified by the face-to-face interview. Poor health rating and longstanding illness were collected through the baseline questionnaire.

### Statistical Analysis

We calculated follow-up time in person-years from the return of the baseline questionnaire to the date of diagnosis cholelithiasis, death, or the end of follow-up, whichever came first. We estimated the hazard ratios (HRs) and 95% confidence intervals (CIs) using Cox regression models. Risk differences (RDs) were calculated based on the incidence rate and HR with the methods described by Altman D.G. (Altman and Andersen, 1999). To address potential reverse causation where symptoms of undiagnosed cholelithiasis may be related to acid suppressants use, we lagged the exposure for 2 years, allowing a time window for the development of cholelithiasis.

In the basic model, we stratified the analyses jointly by age, sex and UK Biobank assessment center. In multivariable adjusted model 1, we additionally adjusted for race (*white* or *other*), BMI (*< 18.5*, *18.5–24.9*, *25–30*, ≥ *30*), index of multiple deprivation (a measure of socio-economic status), smoking status (*never smoker*, *previous smoker*, *current smoker*), alcohol consumption (*never or special occasions only*, *one to three times a month*, *one to four times a week*, *daily or almost daily*), physical activity (*MET < 9 h/week*, *9–27 h/week*, ≥ *27 h/week*, *unknown/missing*), portions of fruit and vegetable intake (*< 5 portions per day*, ≥ *5 portions per day*), clinical indication for gastric acid suppressant drug use (gastro-esophageal reflux disease, gastric or duodenal ulcer, gastrointestinal bleeding), presence of general comorbidities (hypertension, hypercholesterolemia and diabetes), and general health indicator variables, including overall health rating (*poor*, *fair*, *good*, *excellent*) and longstanding illness (*yes* or *no*). To address the possible confounding effect of other medications and supplements, we additionally adjusted for non-steroidal anti-inflammatory drugs (*yes* or *no*), aspirin (*yes* or *no*), statin (*yes* or *no*), angiotensin-converting enzyme inhibitors (*yes* or *no*), angiotensin receptor blocker (*yes* or *no*), beta-blockers (*yes* or *no*), multivitamin use (*yes* or *no*), and mineral supplements intake (*yes* or *no*).

To investigate potential effect modifiers, we conducted stratified analyses according to sex, age, race, body mass index (BMI), smoking status, alcohol intake, dietary quality, and physical activity. To explore genetic interactions, we undertook stratified analysis by gallstone-related single nucleotide polymorphisms (SNPs), including *rs1260326, rs11887534, rs4245791, rs9843304, rs6471717, and rs2547231*. These SNPs have been confirmed as genetic risk factors for gallstone disease by meta-analysis of Genome-Wide Association Studies (GWAS) (Joshi et al., 2016). To identify population with high risk of acid suppressant-associated gallstones, we evaluated the relative (HR) and absolute effect (RD) of acid suppressants on gallstones according to the baseline predicted gallstone risk. We establish predicted models with logistic regression, taking the development of gallstone as the dependent variable and major known genetic (aforementioned SNPs (Joshi et al., 2016)) and environmental risk factors (age, sex, race, BMI, smoking status, alcohol consumption, physical activity, clinical indication for gastric acid suppressant drug use and general health indicator variables (Pak and Lindseth, 2016; Yuan S et al., 2021)) as the independent variables. Three predicted models were established: 1) model only included with genetic variables; 2) model only included with environmental variables; 3) model included both genetic and environmental variables. The baseline predicted risks for individual participants were calculated using the established models. Such risk stratification has been used to identify individuals with high risk of PPI-associated type 2 diabetes (He et al., 2020) and stroke (Yang et al., 2021).

In the sensitivity analyses, we lagged the exposure for an even longer time (4 years). We further conducted a propensity score–matched analysis to check the robustness of the primary results. We applied logistic regression including all covariates mentioned above to estimate the propensity score. Each participant who initiated PPI was matched to whom did not on propensity score by using the greedy nearest neighbor matching algorithm without replacement. The optimal ratio from the analysis of variable multiple pairing was 1:4. Last, we calculated the E-value to test the potential influence of unmeasured confounders. E-value is defined as the minimum strength of association that an unmeasured confounder would need to have with both the exposure and the outcome to fully explain away a specific association (Vanderweele and Ding, 2017). We performed all analyses using SAS software, Version 9.4 (SAS Institute, Cary, North Carolina, United States).

## Results

### Participant Baseline Characteristics

This study included a total of 477,293 participants, of which 45,538 were regular PPI users and 9,596 were regular H2RA users. Table 1 presents the baseline characteristics of included participants. Regular PPI or H2RA users had similar characteristics when compared with non-acid suppressant users. Specifically, they had a higher rate of comorbidities (hypertension, hypercholesterolemia and diabetes), and were more likely to use other medications (aspirin, non-aspirin NSAIDs, and statin). As expected, they had higher rates of esophagitis, GERD, esophageal stricture, gastric or duodenal ulcer, gastritis, upper gastrointestinal tract bleeding, poor health rating, and longstanding illness.

**TABLE 1:** Baseline characteristics of included participants.

	Regular use of PPI		Regular use of H2RA		Overall
	Yes (N = 45,538)	No (N = 431,755)	Yes (N = 9,596)	No (N = 467,697)	(N = 477,293)
Mean (SD) Age, years	60.0 (7.30)	56.6 (8.12)	58.0 (8.01)	56.9 (8.11)	56.9 (8.10)
Female, N (%)	23,683 (52.0)	230,750 (53.4)	4,995 (52.1)	249,438 (53.3)	254,433 (53.3)
White, N (%)	43,248 (95.0)	407,941 (94.5)	9,074 (94.6)	442,115 (94.5)	451,189 (94.5)
Mean (SD) Index of multiple deprivation	20.0 (15.7)	17.0 (13.8)	20.7 (16.3)	17.2 (14.0)	17.3 (14.1)
Mean (SD) BMI, kg/m2	29.0 (5.05)	27.1 (4.64)	28.8 (5.11)	27.3 (4.70)	27.3 (4.71)
Never drinker, N (%)	5,373 (11.8)	32,074 (7.4)	1,031 (10.7)	36,416 (7.8)	37,447 (7.8)
Never smoker, N (%)	21,170 (46.5)	241,984 (56.0)	4,327 (45.1)	258,827 (55.3)	263,154 (55.1)
Median (IQR) physical activity, MET hours/week	41.4 (45.2)	44.7 (45.4)	40.7 (44.5)	44.5 (45.4)	44.4 (45.4)
>5 portions of fruit and vegetable per day, N (%)	17,017 (37.4)	162,360 (37.6)	3,361 (35.0)	176,016 (37.6)	179,377 (37.6)
Hypertension, N (%)	32,519 (71.4)	245,795 (56.9)	6,384 (66.5)	271,930 (58.1)	278,314 (58.3)
Hypercholesterolaemia, N (%)	16,886 (37.1)	70,743 (16.4)	2,838 (29.6)	84,791 (18.1)	87,629 (18.4)
Diabetes, N (%)	5,193 (11.4)	22,257 (5.2)	905 (9.4)	26,545 (5.7)	27,450 (5.8)
Oesophagitis, N (%)	3,636 (8.0)	2,702 (0.6)	387 (4.0)	5,951 (1.3)	6,338 (1.3)
GERD, N (%)	19,946 (43.8)	12,544 (2.9)	3,066 (32.0)	29,424 (6.3)	32,490 (6.8)
Gastric or duodenal ulcer, N (%)	4,536 (10.0)	6,114 (1.4)	913 (9.5)	9,737 (2.1)	10,650 (2.2)
Gastritis, N (%)	8,472 (18.6)	11,163 (2.6)	1,071 (11.2)	18,564 (4.0)	19,635 (4.1)
Upper gastrointestinal tract bleeding, N (%)	1,432 (3.1)	2,260 (0.5)	181 (1.9)	3,511 (0.8)	3,692 (0.8)
Aspirin use, N (%)	11,352 (24.9)	56,306 (13.0)	2034 (21.2)	65,624 (14.0)	67,658 (14.2)
Non-aspirin NSAIDs use, N (%)	7,706 (16.9)	69,274 (16.0)	2,300 (24.0)	74,680 (16.0)	76,980 (16.1)
Statin use, N (%)	15,134 (33.2)	61,454 (14.2)	2,429 (25.3)	74,159 (15.9)	76,588 (16.0)
Poor health rating, N (%)	6,309 (13.9)	14,342 (3.3)	1,147 (12.0)	19,504 (4.2)	20,651 (4.3)
Longstanding illness, N (%)	27,017 (59.3)	121,652 (28.2)	4,930 (51.4)	143,739 (30.7)	148,669 (31.1)

### Regular PPI/H2RA Use and Risk of Cholelithiasis

Table 2 indicates the association between regular PPI/H2RA use and risk of cholelithiasis. We identified 12,870 cases of cholelithiasis over a median follow-up of 8.1 years. The incidence of cholelithiasis tended to be higher in regular users of acid suppressants than non-users (PPI: 491.2 vs. 219 per 1,000 person-years, H2RA: 438.6 vs. 240.4 per 1,000 person-years). Regular use of PPIs showed a positive association with cholelithiasis. In the basic model, regular use of PPIs was significantly associated with a higher risk of cholelithiasis (HR 2.03, 95% CI 1.94–2.12). This association was attenuated somewhat, but remained significant after multivariable adjustment for demographic factors, lifestyle habits, and PPI clinical indications (HR 1.22, 95% CI 1.16–1.29), and showed no change in the estimated effect after further adjustment for other commonly used medications. Regular use of H2RA also showed an increased risk of cholelithiasis, with a fully-adjusted HR of 1.16 (95% CI, 1.05–1.28). The absolute risk increased by 3.39 per 1,000 person-years (95% CI 2.81–3.86) for PPIs and 2.55 per 1,000 person-years (95% CI 1.04–3.43) for H2RAs.

**TABLE 2:** Associations between regular PPI/H2RA use and risk of cholelithiasis.

	Regular PPI user		Regular H2RA user	
	No	Yes	No	Yes
Cases	10,506	2,364	12,420	450
Person-years	4,787,178	481,232	5,165,809	102,600
Incidence rate ^a^	2.20	4.91	2.40	4.39
Hazard Ratio (95% CI)				
Crude model^b^	1.00 (Ref)	2.03 (1.94,2.12)	1.00 (Ref)	1.75 (1.59,1.92)
Multivariate-adjusted model 1^c^	1.00 (Ref)	1.22 (1.16,1.29)	1.00 (Ref)	1.17 (1.06,1.29)
Multivariate-adjusted model 2^d^	1.00 (Ref)	1.22 (1.16,1.29)	1.00 (Ref)	1.16 (1.05,1.28)
Risk Difference (95% CI)				
Crude model^b^	1.00 (Ref)	4.89 (4.88, 4.90)	1.00 (Ref)	4.31 (4.21, 4.36)
Multivariate-adjusted model 1^c^	1.00 (Ref)	3.39 (2.81, 3.86)	1.00 (Ref)	2.64 (1.22, 3.48)
Multivariate-adjusted model 2^d^	1.00 (Ref)	3.39 (2.81, 3.86)	1.00 (Ref)	2.55 (1.04, 3.43)

a Per 1,000 person years.

b Crude model: basic Cox proportional hazards model stratified by age, sex and UK, Biobank assessment centre.

c Multivariable adjusted model 1: additionally adjusted for race (*white* or *other*), BMI (<*18.5*, *18.5–24.9*, *25–30*, ≥*30*), index of multiple deprivation, smoking status (*never smoker*, *previous smoker*, *current smoker*), alcohol consumption (*never or special occasions only*, *one to three times a month*, *one to four times a week*, *daily or almost daily*), physical activity (*MET < 9 h/week*, *9–27 h/week*, ≥ *27 h/week*, *unknown/missing*), portions of fruit and vegetable intake (*< 5 portions per day*, ≥ *5 portions per day*), clinical indication for gastric acid suppressant drug use (gastro-oesophageal reflux disease, gastric or duodenal ulcer, gastrointestinal bleeding), presence of general comorbidities (hypertension, hypercholesterolaemia and diabetes), and general health indicator variables, including overall health rating (*poor*, *fair*, *good*, *excellent*) and longstanding illness (*yes* or *no*).

d Multivariable adjusted model 2: additionally adjusted for regular use of other medications (non-steroidal anti-inflammatory drugs, aspirin, statin, angiotensin-converting enzyme inhibitors, angiotensin receptor blocker, beta-blockers), multivitamin use (*yes* or *no*), and mineral supplements intake (*yes* or *no*).

In the sensitivity analyses, we observed no major changes in the associations between PPI/H2RA use and cholelithiasis risk after lagging the exposure for 4 years (Supplementary Table S1). PPI (HR 1.37, 95% CIs 1.30–1.45) and H2RA (HR 1.19, 95% CIs 1.07–1.33) use were still associated with increased risk of cholelithiasis after propensity score matching (Supplementary Table S2). In the analysis for unmeasured confounders, we obtained an E-value of 1.74 for PPIs and 1.59 for H2RAs. Additional adjustment for *H. pylori* eradication or genetic confounding factors did not show major changes in the primary results (Supplementary Tables S3, S4).

Table 3 presented the effects of individual class of PPI/H2RA on the risk of cholelithiasis. For PPIs, there were no major differences between individual types of PPIs. Significant associations were observed for omeprazole, lansoprazole, and esomeprazole. Ranitidine is the primary H2RA used among included participants and had a significantly increased risk (HR 1.14, 95% CI 1.03–1.26). The number of events for other classes of H2RA were too small to estimate the effects.

**TABLE 3:** The associations between regular use of individual class of PPI/H2RA and risk of cholelithiasis.

	Cases	Person-years	Incidence rate^a^	HR (95%CI)^b^	RD (95%CI)^b^
Regular use of PPIs					
Non-PPI user	10,506	4,787,178	2.20	1.00 (Ref)	1.00 (Ref)
Omeprazole user	1,505	310,671	4.84	1.20 (1.13,1.28)	3.18 (2.42, 3.76)
Lansoprazole user	829	170,092	4.87	1.12 (1.03,1.21)	2.30 (0.72, 3.28)
Esomeprazole user	132	22,617	5.84	1.26 (1.06,1.51)	4.30 (1.55, 5.41)
Other PPI user	111	20,282	5.47	1.16 (0.96,1.41)	3.09 (−1.27, 4.83)
Regular use of H2RAs					
Non-H2RA user	12,420	5,165,809	2.40	1.00 (Ref)	1.00 (Ref)
Ranitidine user	422	98,042	4.30	1.14 (1.03,1.26)	2.30 (0.65, 3.26)
Other H2RA user	30	5,352	5.61	1.28 (0.90,1.84)	4.29 (−3.81, 5.53)

a Per 1,000 person years.

b Estimated effects were based on the fully adjusted model (see the footnote in Table 2).

### Subgroup Analysis

Supplementary Table S5 and Supplementary Table S6 showed the subgroup analyses by environmental and genetic factors. The estimated risk of cholelithiasis among PPI users did not differ by age, obesity, smoking status, alcohol intake, physical activity, and fruit and vegetable intake. The PPI-associated cholelithiasis was more evident in males than females (*p* = 0.01) and in ever smokers than non-smokers (*p* = 0.006). In the analyses for potential genetic interactions, we observed no effect with known gallstone-related single nucleotide polymorphisms (SNPs), including rs1260326, rs11887534, rs4245791, rs9843304, rs6471717, and rs2547231.

### Risk Stratification

We undertook additional interaction analyses by baseline gallstone risk, which were determined by genetic risk score, or environmental risk score, or combined risk score integrating genetic and environmental factors (Table 4). The environmental risk score seems to discriminate the participants better than the genetic risk score. The relative risks (indicated by HRs) were similar across various risk strata. However, for PPI users, the absolute risks (indicated by RDs) increased from 1.15 per 1,000 person-years in quartile 1 to 4.09 per 1,000 person-years in quartile four in the analysis by environmental risk score. Stratified analyses by environmental and genetic risk score did not show further improvement in the ability to identify individuals with high PPI-associated cholelithiasis risk (RD is Q1 vs. Q4: 1.37 vs 4.29 per 1,000 person-years).

**TABLE 4:** Risk stratification by predicted genetic or environmental risk score for cholelithiasis at baseline.

	Regular use of PPI					Regular use of H2RA				
	Cases	Person-years	Incidence rate^a^	HR (95%CI)^b^	RD (95%CI)^b^	Cases	Person-years	Incidence rate^a^	HR (95%CI)^b^	RD (95% CI)^b^
**Stratified analysis by genetic risk score**										
Q1	455	114,981	3.96	1.32 (1.16,1.50)	3.28 (2.32, 3.71)	66	23,634	2.79	0.93 (0.72,1.20)	-1.42 (-11.7, 1.93)
Q2	531	118,062	4.50	1.29 (1.14,1.45)	3.56 (2.39, 4.10)	93	24,988	3.72	1.13 (0.91,1.40)	1.92 (-2.44, 3.32)
Q3	568	113,913	5.00	1.34 (1.20,1.50)	4.16 (3.26, 4.63)	120	24,772	4.84	1.31 (1.08,1.59)	3.92 (1.68, 4.64)
Q4	690	115,525	6.00	1.16 (1.04,1.28)	3.34 (1.11, 4.55)	151	25,490	5.92	1.23 (1.04,1.46)	4.10 (1.10, 5.36)
P interaction				0.223	0.337				0.15	0.288
**Stratified analysis by environmental risk score**										
Q1	21	17,533	1.20	1.49 (0.95, 2.32)	1.15 (-0.48, 1.2)	7	9,058	0.77	0.92 (0.4, 2.07)	-0.6 (-56.18, 0.77)
Q2	131	47,353	2.77	1.65 (1.35, 2.01)	2.71 (2.41, 2.76)	38	14,558	2.61	1.62 (1.16, 2.25)	2.55 (1.60, 2.61)
Q3	353	110,383	3.20	1.31 (1.16, 1.49)	2.66 (1.92, 3.01)	80	25,467	3.14	1.2 (0.95, 1.51)	2.15 (-1.05, 2.98)
Q4	1759	290,297	6.06	1.22 (1.15, 1.31)	4.09 (3.24, 4.81)	308	50,404	6.11	1.14 (1.01, 1.28)	3.12 (0.30, 4.64)
P interaction				0.068	<0.001				0.362	0.926
**Stratified analysis by environmental and genetic risk score**										
Q1	29	21,036	1.38	1.72 (1.16, 2.55)	1.37 (0.90, 1.38)	6	9,422	0.64	0.92 (0.41, 2.07)	-0.51 (-48.31, 0.64)
Q2	134	53,495	2.51	1.72 (1.16, 2.55)	2.47 (1.54, 2.50)	35	15,704	2.23	1.43 (1.01, 2.03)	2.07 (0.13, 2.22)
Q3	375	113,935	3.29	1.34 (1.18, 1.52)	2.82 (2.12, 3.12)	96	25,483	3.77	1.42 (1.15, 1.76)	3.41 (2.14, 3.71)
Q4	1706	274,015	6.23	1.23 (1.15, 1.31)	4.29 (3.32, 4.94)	293	48,274	6.07	1.1 (0.97, 1.24)	2.43 (-1.00, 4.29)
P interaction				0.191	<0.001				0.176	0.237

a Per 100,000 person years.

b Estimated effects were based on the fully adjusted model (see the footnote in Table 2).

## Discussion

This analysis of an ongoing cohort including 0.47 million participants showed that regular use of acid suppressants was associated with an increased risk of cholelithiasis, with an absolute risk of 3.39 per 1,000 person-years for PPIs and 2.55 per 1,000 person-years for H2RAs. The associations were similar among individual type of PPIs. The PPI associated absolute risk was increased with the baseline gallstone risk, which could be easily measured by demographic and lifestyle information.

Currently, no epidemiological study have directly evaluated the use of acid suppressants and risk of cholelithiasis. Cholelithiasis is responsible for 90–95% of cases of acute cholecystitis (Kimura et al., 2007). A recent case-control study based on National Health Insurance Research Database in Taiwan showed that PPI use was associated with acute cholecystitis as compared with control (odds ratio 1.23, 95%CI 1.13–1.34) (Chuang et al., 2019). This study is a retrospective study, and the results may be influenced by inadequate assessment of exposure and insufficient adjustment of important confounders such as BMI, lifestyle habits and indications of PPI therapy. In a large nationwide cohort study, Min et al. reported that PPI use was associated with an increased risk of cholangitis (HR 5.75; 95% CI, 4.39–7.54) (Min et al., 2019). A propensity score matching analysis showed that PPI use was associated with significantly higher risk of recurrence of common bile duct stones in patients treated with endoscopic sphincterotomy (Fukuba et al., 2017). Additionally, in a hospital-based case-control study from China, PPI use was associated with an increased risk of gallbladder cancer, with higher risk observed in patients with longer duration of PPIs use (Xiong et al., 2020). A recent Swedish population-based cohort study showed that PPI use increases the risk of incident biliary tract carcinoma (SIRs 1.58, 95% CI 1.37–1.81) over a median follow-up of 5.3 years (Kamal et al., 2021). These findings were in line with the observed association in the present analysis, because gallstone is a proven risk factor for gallbladder cancer (Hundal and Shaffer, 2014; Nogueira et al., 2014). In one of our ongoing projects based on the Nurse Health Study II dataset, the preliminary analyses also suggested that regular use of PPIs was associated with a significantly increased gallstone risk (data not published).

The exact mechanism underlying the association between acid suppression treatment and cholelithiasis is still unclear. A potential explanation is that PPIs reduce gallbladder function, which in turn increase the development of gallstones. It has been shown that PPIs could delay the gastric emptying, increase the concentration of bile in the gastric fluid, so lead to a decrease in cholecystokinin release (Rasmussen et al., 1997; Foltz et al., 2015). In a small cohort of 19 healthy volunteers, 15 subjects showed a reduced gallbladder motility after 30 days of antisecretory therapy (omeprazole 40 mg daily) (Cahan et al., 2006). Gallbladder emptying is reduced and, thus, facilitates stone formation (Pauletzki et al., 1996). Additionally, PPIs may increase the cholelithiasis risk through intestinal flora imbalance and bile acid metabolism (Wang et al., 2020). Strong evidence showed that PPI use could lead to major changes in the microbial profile, for example, reduced abundance of *Bifidobacterium*, and *Ruminococcoceae* (Imhann et al., 2016). A recent study showed that after long-term PPI exposure, the fecal microbial profile in mice was altered and showed similarity to those taking a high-fat diet (Yang et al., 2020), which is often used to induce gallstones in mouse models. Many of these altered gut bacteria, such as *Bifidobacterium*, express bile salt hydrolase (BSH) enzymatic activity, which works as a gateway reaction in the metabolism of bile acids (BAs) (Foley et al., 2019). They may deconjugate tauroconjugated or glycoconjugated primary BAs to produce unconjugated counterparts, which can be further metabolized to secondary and tertiary BAs by 7a dehydroxylase enymes (Joyce et al., 2014). The increase in the concentration of secondary bile acids promotes gallstone formation (Wang et al., 2020). Lastly, long-term use of PPIs may result in ductal epithelial proliferation, micropapillary growth of biliary epithelium, focal bile duct stricture formation, and bile duct obstruction (Yang et al., 2020). These morphological changes further facilitate the formation of gallstones.

## Strengths and Limitations of This Study

A strength of this study is the large sample size and case number, which enable us to precisely estimate the association. Second, the cohort design reduced potential recall and selection biases. Thirdly, we comprehensively investigated the interaction effects with environmental and genetic factors. Last, robust sensitivity analyses additionally increased our confidence in the results.

This study also has its limitations. First, as an observational study, we cannot exclude the residual confounding effect. However, in our sensitivity analysis, we got an E-value of 1.74; As we have comprehensively adjusted for major risk factors of gallstone, there is unlikely an unknown confounder showing HRs with both PPI use and gallstone risk of over 1.74. Second, we did not have information of the dosage and frequency of PPI and H2RA, so we could not conduct further analysis for those factors. Thirdly, the observed associations maybe confounded by the indications of PPIs and H2RAs; However, the association persisted after adjustment for the indications and restriction in participants with indications. Lastly, misclassification of exposure during follow-up might exist because PPI/H2RA use was only evaluated once at baseline. However, misclassification would underestimate the true effects as there were many PPI/H2RA users (with high risk) in the control group.

## Conclusion

Overall, this prospective cohort study suggested regular use of acid suppression drugs was likely to be associated with an increased risk of cholelithiasis. Physicians should be aware of this association when prescribing these medicines, particularly for individuals requiring long-term treatment and are already at high risk of cholelithiasis. An evaluation of baseline predicted cholelithiasis risk based on known risk factors may contribute to individualized use of PPIs. Due to the fact that the use of acid suppressants is common and the incidence of cholelithiasis is high, the potential impact on public health could be substantial if the association is causal. Further prospective cohort studies or secondary analysis of randomized trials are needed to confirm the causal relationship. Investigation of the mechanisms behind this association is also necessary to implement prevention of acid suppression-related cholelithiasis.

## Data Availability

The data that support the findings of this study are available from the UK Biobank (application number 51671, approved August 2019) but restrictions apply to the availability of these data, which were used under license for the current study. The R code for data analysis is available by contacting the corresponding authors.
