# Supplementary material for: Association Between Regular Use of Gastric Acid Suppressants and Subsequent Risk of Cholelithiasis: A Prospective Cohort Study of 0.47 Million Participants
Source: Front Pharmacol. 2022 Jan 28;12:813587. doi: 10.3389/fphar.2021.813587 (PMC8831324; doi:10.3389/fphar.2021.813587)
Supplement: Supplementary file 1 [file Presentation1.pdf]

Supplementary Material

1. Supplementary Figure

Figure S1. Flowchart of participant selection.

2. Supplementary Tables

Supplementary Table 1 Sensitivity analyses of the association between acid suppressant use and risk of cholelithiasis after lagging the exposure for 4 years.

Supplementary Table 2 Sensitivity analyses of the association between acid suppressant use and risk of cholelithiasis using propensity score – matching approach in the UK biobank

Supplementary Table 3 Sensitivity analyses of the association between acid suppressant use and risk of cholelithiasis after further adjustment for *H. pylori* eradication in the UK biobank

Supplementary Table 4 Sensitivity analyses of the association between acid suppressant use and risk of cholelithiasis by adjusting for genetic confounding.

Supplementary Table 5 Stratified analyses of the associations between acid suppressant use and risk of cholelithiasis by environmental factors

Supplementary Table 6 Genetic interaction of regular use of PPIS/H2RAs with cholelithiasis

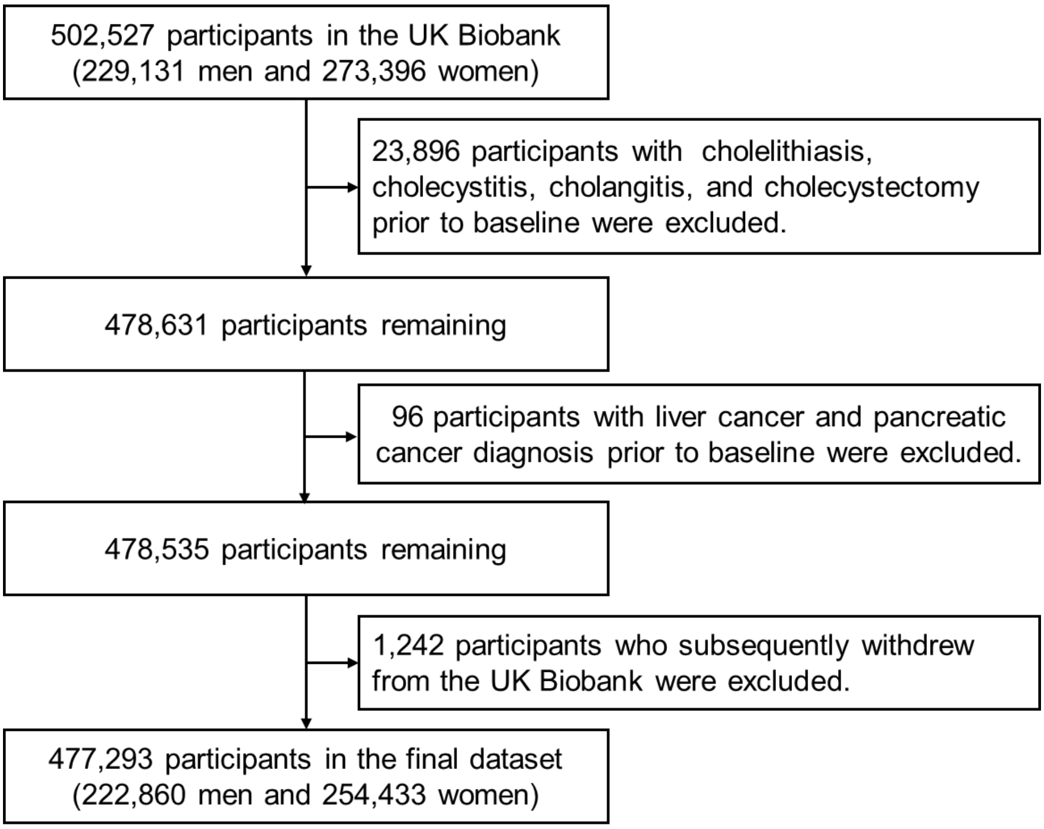

Figure S1. Flowchart of participant selection.

Table S1. Sensitivity analyses of the association between acid suppressant use and risk of cholelithiasis after lagging the exposure for 4 years.

	Non-regular PPI user	Regular PPI user	Non-regular H2RA user	Regular H2RA user
Cases	8387	1826	9866	347
Person-years	4771884	477647	5147572	101959
Incidence rate*	1.76	3.82	1.92	3.40
HR [95%CI] †	1.00 [Ref]	1.23 [1.16 ,1.31]	1.00 [Ref]	1.14 [1.02 ,1.27]
RD [95%CI] †	1.00 [Ref]	2.76 [2.25, 3.14]	1.00 [Ref]	1.87 [0.37, 2.67]

* Per 1000 person years.

† Estimated effects were based on the fully adjusted model.

Table S2. Sensitivity analyses of the association between acid suppressant use and risk of cholelithiasis using propensity score–matching approach in the UK biobank.

	Non-regular PPI user	Regular PPI user	Non-regular H2RA user	Regular H2RA user
Cases	3873	2120	1483	438
Person-years	1121046	442871	373443	107762
Incidence rate*	3.46	4.79	3.97	4.07
HR [95%CI] †	1.00 [Ref]	1.37 [1.30 ,1.45]	1.00 [Ref]	1.19 [1.07 ,1.33]
RD [95%CI] †	1.00 [Ref]	4.12 [3.82, 4.35]	1.00 [Ref]	2.64 [1.3, 3.4]

* Per 1000 person years.

† Estimated effects were based on the fully adjusted model.

Table S3. Sensitivity analyses of the association between acid suppressant use and risk of cholelithiasis after adjustment for H. pylori eradication

	Non-regular PPI user	Regular PPI user	Non-regular H2RA user	Regular H2RA user
Cases	10506	2364	12420	450
Person-years	4787178	481232	5165809	102600
Incidence rate*	2.20	4.91	2.40	4.39
HR [95%CI] †	1.00 [Ref]	1.21[1.15, 1.29]	1.00 [Ref]	1.16 [1.05 ,1.28]
RD [95%CI] †	1.00 [Ref]	3.30 [2.70, 3.86]	1.00 [Ref]	2.55 [1.04, 3.43]

* Per 1000 person years.

† Estimated effects were based on the fully adjusted model.

Table S4. Sensitivity analyses of the association between acid suppressant use and risk of cholelithiasis by adjusting for genetic confounding.

	Non-regular PPI user	Regular PPI user	Non-regular H2RA user	Regular H2RA user
Cases	3873	2120	1483	438
Person-years	1121046	442871	373443	107762
Incidence rate*	3.46	4.79	3.97	4.07
HR [95%CI] †	1.00 [Ref]	1.37 [1.30 ,1.45]	1.00 [Ref]	1.19 [1.07 ,1.33]
RD [95%CI] †	1.00 [Ref]	4.12 [3.82, 4.35]	1.00 [Ref]	2.64 [1.3, 3.4]

* Per 1000 person years.

† Estimated effects were based on the fully adjusted model.

Table S5. Stratified analyses of the associations between acid suppressant use and risk of cholelithiasis by environmental factors

	Regular use of PPI					Regular use of H2RA				
	Cases	Person-years	Incidence rate *	Multivariate-adjusted HR (95%CI) †	P interaction	Cases	Person-years	Incidence rate *	Multivariate-adjusted HR (95%CI) †	P interaction
**Sex**										
Male	1423	251506	5.66	1.33 [1.22 ,1.45]	0.010	274	53575	5.11	1.22 [1.05 ,1.43]	0.316
Female	941	229726	4.10	1.15 [1.07 ,1.23]		176	49025	3.59	1.11 [0.98 ,1.26]	
**Age**										
< 60	901	203105	4.44	1.14 [1.04 ,1.24]	0.807	217	54621	3.97	1.20 [1.04 ,1.38]	0.249
≥ 60	1463	278127	5.26	1.30 [1.21 ,1.39]		233	47979	4.86	1.12 [0.98 ,1.28]	
**Obesity**										
No	1193	308738	3.86	1.19 [1.11 ,1.29]	0.38	236	67405	3.50	1.19 [1.04 ,1.36]	0.48
Yes	1171	172494	6.79	1.25 [1.15 ,1.35]		214	35195	6.08	1.11 [0.97 ,1.28]	
**Never smoker**										
Yes	1005	227192	4.42	1.12 [1.03 ,1.21]	0.006	198	46755	4.24	1.17 [1.01 ,1.36]	0.844
No	1359	254041	5.35	1.31 [1.22 ,1.42]		252	55846	4.51	1.15 [1.01 ,1.31]	
**Never drinker**										
Yes	370	56627	6.53	1.23 [1.06 ,1.42]	0.437	61	10734	5.68	1.09 [0.83 ,1.42]	0.502
No	1994	424606	4.70	1.22 [1.15 ,1.3]		389	91867	4.23	1.16 [1.05 ,1.29]	
**Physical activity**										
< median	1677	312131	5.37	1.27 [1.19 ,1.36]	0.899	327	65969	4.96	1.22 [1.09 ,1.37]	0.301
≥ median	687	169101	4.06	1.12 [1.02 ,1.24]		123	36632	3.36	1.03 [0.86 ,1.24]	
**Below 5 portions of fruit and vegetable per day**										
Yes	1525	300038	5.08	1.23 [1.15 ,1.32]	0.593	297	66411	4.47	1.18 [1.04 ,1.33]	0.709
No	839	181194	0.35	1.21 [1.10 ,1.32]		153	36190	0.35	1.13 [0.95 ,1.33]	

* Per 1000 person years.

† Estimated effects were based on the fully adjusted model.

Table S6. Genetic interaction of regular use of PPIS/H2RAs with cholelithiasis

	Regular use of PPIs					Regular use of H2RAs				
	**Cases**	**Person-years**	**Incidence rate**	**Multivariate-adjusted HR [95% CI]**	**Multivariate-adjusted RD [95% CI]**	**Cases**	**Person-years**	**Incidence rate**	**Multivariate-adjusted HR [95% CI]**	**Multivariate-adjusted RD [95% CI]**
**rs1260326**										
CC	863	171515	5.03	1.26 [1.15 ,1.38]	3.76 [2.76, 4.36]	174	37451	4.65	1.23 [1.05 ,1.44]	3.30 [1.09, 4.21]
CT	1048	218781	4.79	1.25 [1.15 ,1.36]	3.53 [2.64, 4.09]	196	46457	4.22	1.12 [0.97 ,1.30]	2.03 [-0.75, 3.40]
TT	340	73008	4.66	1.36 [1.18 ,1.58]	3.98 [2.89, 4.45]	61	15136	4.03	1.19 [0.92 ,1.56]	2.62 [-2.23, 3.85]
P interaction				0.507	0.709				0.762	0.626
**rs11887534**										
CC	19	1666	11.41	1.23 [0.49 ,3.09]	7.33 [-100, 11.4]	3	378	7.94	0.19 [0.03 ,1.11]	-384.62 [-833.33, 3.27]
CG	355	52997	6.70	1.18 [1.02 ,1.36]	3.98 [0.64, 5.59]	88	11960	7.36	1.40 [1.12 ,1.75]	6.33 [3.28, 7.17]
GG	1890	410902	4.60	1.29 [1.21 ,1.37]	3.63 [3.11, 3.97]	342	87150	3.92	1.13 [1.01 ,1.26]	2.01 [0.21, 2.99]
P interaction				0.517	0.955				0.502	＜0.001
**rs4245791**										
TT	1142	213033	5.36	1.27 [1.17 ,1.37]	4.05 [3.16, 4.59]	227	46226	4.91	1.18 [1.03 ,1.35]	3.02 [0.72, 4.15]
TC	909	202078	4.50	1.26 [1.15 ,1.37]	3.39 [2.50, 3.89]	171	42690	4.01	1.16 [0.99 ,1.36]	2.35 [-0.23, 3.46]
CC	206	49632	4.15	1.28 [1.05 ,1.56]	3.26 [1.00, 3.96]	34	10412	3.27	1.00 [0.70 ,1.44]	NA
P interaction				0.272	0.367				0.946	0.602
**rs9843304**										
TT	645	139409	4.63	1.23 [1.11 ,1.37]	3.28 [2.07, 3.99]	120	30164	3.98	1.11 [0.92 ,1.34]	1.81 [-2.21, 3.37]
TC	1102	227202	4.85	1.28 [1.18 ,1.39]	3.76 [2.99, 4.24]	214	48000	4.46	1.19 [1.03 ,1.37]	2.86 [0.67, 3.86]
CC	517	98954	5.23	1.29 [1.15 ,1.46]	4.09 [2.85, 4.76]	99	21323	4.64	1.23 [0.99 ,1.51]	3.29 [-0.26, 4.34]
P interaction				0.627	0.497				0.743	0.718
**rs6471717**										
AA	977	204917	4.77	1.33 [1.22 ,1.46]	3.95 [3.30, 4.36]	169	43980	3.84	1.08 [0.92 ,1.26]	1.38 [-2.15, 2.94]
AG	995	207356	4.80	1.20 [1.10 ,1.31]	3.15 [1.99, 3.88]	202	43655	4.63	1.28 [1.10 ,1.47]	3.60[1.92, 4.26]

GG	292	53292	5.48	1.34 [1.14 ,1.57]	4.55 [2.84, 5.20]	62	11852	5.23	1.16 [0.88 ,1.53]	2.97 [-4.60, 4.91]
P interaction				0.267	0.166				0.400	0.298
**rs2547231**										
AA	1641	325376	5.04	1.25 [1.17 ,1.34]	3.70 [2.99, 4.21]	314	69639	4.51	1.17 [1.04 ,1.31]	2.71 [0.88, 3.66]
AC	579	127553	4.54	1.33 [1.19 ,1.49]	3.77 [2.91, 4.22]	107	27270	3.92	1.15 [0.94 ,1.40]	2.21 [-1.55, 3.50]
CC	44	12637	3.48	1.03 [0.67 ,1.58]	0.54 [-19.05, 3.35]	12	2579	4.65	1.59 [0.83 ,3.05]	4.46 [-6.94, 4.65]
P interaction				0.978	0.846				0.668	0.780

* Per 1000 person years.

† Estimated effects were based on the fully adjusted model.
