# Supplementary material for: Association Between Regular Use of Gastric Acid Suppressants and Subsequent Risk of Cholelithiasis: A Prospective Cohort Study of 0.47 Million Participants
Source: Front Pharmacol. 2022 Jan 28;12:813587. doi: 10.3389/fphar.2021.813587 (PMC8831324; doi:10.3389/fphar.2021.813587)
Supplement: Supplementary file 2 [file DataSheet1.PDF]

```
############################调用---------------------------------
library(survminer)
library(car)
library(infotheo)
library(survival)
library(ggplot2)
library(rms)
library(table1)
library(dplyr)
library(tidyverse)
library(ggsci)
library("survival")
library("survminer")
library(effects)
library(matrixStats)
library(dplyr)
library(table1)
library(survival)
library(lattice)
library(Formula)
library(grid)
library(magrittr)
library(checkmate)
library(Rcpp)
library(htmlTable)
library(Gmisc)
library(Rcpp)
library(htmlTable)
library(forestplot)
library(Greg)
library(ggplot2)
library(SparseM)
library(Hmisc)
library(rms)
library(infotheo)
library(sysfonts)
library(showtextdb)
library(cowplot)
library(showtext)
library(psych)
library(reshape)
##导入数据
library(haven)
###导入数据------------------------------
ukb<-read.csv("D:/biobank/usedata/analyses.csv",header=TRUE, sep=",")
biobank<-ukb

# survtime="survtime of cancer"
# Survtime_d="survtime of death"
```

```
# Survtime_L="Date lost to follow-up"

###数据初步整理，加标签------------
table(biobank$income)
biobank$income<-factor(biobank$income,levels =c( "Less than 18,000","18,000 to 30,999","31,000 to 51,999"
                                                  ,"52,000 to 100,000", "Greater than 100,000", "99"),
                        labels =c( "Less than 18,000","18,000 to 30,999","31,000 to 51,999"
                                   ,"52,000 to 100,000", "Greater than 100,000", "unkonwn/missing"))

biobank$edu<-factor(biobank$edu,levels = c("College or University degree" ,"A levels/AS levels or equivalent"
                                           ,"O levels/GCSEs or equivalent" ,"CSEs or equivalent" ,"NVQ or HND or HNC or equivalent"
                                           ,"Other professional qualifications eg: nursing, teaching" ,"99"),
                    labels = c("College or University degree" ,"A levels/AS levels or equivalent"
                               ,"O levels/GCSEs or equivalent" ,"CSEs or equivalent" ,"NVQ or HND or HNC or equivalent"
                               ,"Other professional qualifications eg: nursing, teaching" ,"unkonwn/missing"))
table(biobank$eth)
biobank$eth<-factor(biobank$eth,levels = c("1" ,"2" ,"3" ,"4","5" ,"6", "99" ), labels=c("white" ,"Mixed" ,"Asian or Asian British" ,"Black or Black
British"
                                                                                                 ,"Chinese" ,"other",
"unkonwn/missing" ))
table(biobank$salt)
biobank$salt<-factor(biobank$salt,levels = c("Never/rarely" ,"Sometimes" ,"Usually" ,"Always" ,"99" )
                     ,labels = c("Never/rarely" ,"Sometimes" ,"Usually" ,"Always" ,"unkonwn/missing" ))

table(biobank$drinking)
biobank$drinking<-factor(biobank$drinking,levels = c("Daily or almost daily" ,"Three or four times a week" ,"Once or twice a week"
                                                     ,"One to three times a month" ,"Special occasions only" ,"Never" ,"99" )
                         ,labels = c("Daily or almost daily" ,"Three or four times a week" ,"Once or twice a week"
                                     ,"One to three times a month" ,"Special occasions only" ,"Never" ,"unkonwn/missing" ))

table(biobank$smoking)
biobank$smoking<-factor(biobank$smoking,levels = c("Current" ,"Previous"    ,"Never" ,"99"),labels =
c("Current" ,"Previous"    ,"Never" ,"unkonwn/missing"))
biobank$Phy_act <-biobank$MET_g
table(biobank$Phy_act)
biobank$Phy_act<-factor(biobank$Phy_act,levels = c("low","moderate" ,"high" ,"99"),labels =
c("low","moderate" ,"high" ,"unkonwn/missing"))
table(biobank$hp)
biobank$hp<-factor(biobank$hp,levels = c(0,1),labels=c("No", "Yes"))
#fft<-which(colnames(biobank)=="gibl_s")
#ffe<-which(colnames(biobank)=="db_s")
#for (i in c(fft:ffe)){    biobank[,i]<-factor(biobank[,i],levels = c("No","Yes" ))}


biobank$fruit_g<-factor(biobank$fruit_g,levels = c(0,1,99),labels = c("No","Yes" ,"Missing"))
#####注意变量的位置，数据修改后，变量位置也要修改；
# for death
death=which(colnames(biobank)=="death")
LH_d=which(colnames(biobank)=="LH_d")
```

```
for (i in c(death:LH_d)){
    biobank[,i]<-factor(biobank[,i],levels = c(0,1),labels=c("No", "Yes"))}



biobank$juejin<-NA
biobank$juejin[biobank$menopause=="Yes"]<-1
biobank$juejin[biobank$menopause=="No"]<-2
biobank$juejin[biobank$menopause!="No"&biobank$menopause!="Yes"&biobank$gender=="Female"]<-3
biobank$juejin[biobank$juejin==3 &biobank$age>=53]<-1
biobank$juejin[biobank$juejin==3]<-2
biobank$juejin<-factor(biobank$juejin,levels = c(1,2),labels=c( "postmenopause","premenopause"))
table(biobank$juejin, biobank$breast_c)

###For IBS related disorders
lll=which(colnames(biobank)=="IBS_in")
aaa=which(colnames(biobank)=="caesarian")
for (i in c(lll:aaa)){
    biobank[,i]<-factor(biobank[,i],levels = c(0,1),labels=c("No", "Yes"))}
biobank$IBS_s<-factor(biobank$IBS_s,levels = c(0,1,2,99),labels=c("No","Started suddenly", "Came on gradually","Cannot remember"))

### for some medcine
lll=which(colnames(biobank)=="niza2")
aaa=which(colnames(biobank)=="coeliac")
for (i in c(lll:aaa)){
    biobank[,i]<-factor(biobank[,i],levels = c(0,1),labels=c("No", "Yes"))}


lll=which(colnames(biobank)=="lanso0")            #lanso0="lansoprazole at baseline"
aaa=which(colnames(biobank)=="cime2")
for (i in c(lll:aaa)){
    biobank[,i]<-factor(biobank[,i],levels = c(0,1),labels=c("No", "Yes"))}


biobank$Laxat<-factor(biobank$Laxat,levels=c(0,1,99),labels = c("No","Yes" ,"Missing"))
biobank$vitamin<-factor(biobank$vitamin,levels=c(0,1,99),labels = c("No","Yes" ,"Missing"))
biobank$mineral<-factor(biobank$mineral,levels =c(0,1,99),labels= c("No","Yes" ,"Missing"))
biobank$ASP<-factor(biobank$ASP)
biobank$Par<-factor(biobank$Par)
biobank$Ibu<-factor(biobank$Ibu)
biobank$NASIDS<-factor(biobank$NASIDS)



##for MetS
####cob 为"cen_obesity"的位置，MTN 为"MetS_c"  的位置
cob=which(colnames(biobank)=="cen_obesity")
MTN=which(colnames(biobank)=="MetS_c")
for (i in c(cob:MTN)){
    biobank[,i]<-factor(biobank[,i],levels = c(0,1),labels=c("No", "Yes"))}
```

```
biobank$MetS_c4<-as.factor(biobank$MetS_c4)



#######---------end--初步整理完毕------------------
###对数据进一步处理---------------生成 dataukb-----------------
dataukb<-biobank
level_key <-c("Mixed" ="Non-White","Asian or Asian British"="Non-White" ,"Black or Black British"="Non-White",
                        "Chinese" ="Non-White","other"="Non-White")
dataukb$eth<-recode_factor(dataukb$eth,!!!level_key)
level_ke <-c("Three or four times a week"="1-4 times a week" ,"Once or twice a week"="1-4 times a week",
                      "Special occasions only"="Special occasions only or never" ,"Never"="Special occasions only or never")
dataukb$drinking4<-recode_factor(dataukb$drinking,!!!level_ke)
dataukb$drinking4<-factor(dataukb$drinking4,levels = c("Daily or almost daily" ,
                                                                                                "1-4 times a week" ,
                                                                                                "One to three times a month" ,
                                                                                                "Special occasions only or never" ,
                                                                                                "unkonwn/missing" ))

dataukb<-within(dataukb,
                           {drink<-NA
                           drink[drinking=="Never"]=0
                           drink[drinking=="unkonwn/missing" |drinking=="Special occasions only"]=1
                           drink[drinking=="One to three times a month"]=2
                           drink[drinking=="Once or twice a week" |drinking=="Three or four times a week"]=3
                           drink[drinking=="Daily or almost daily"]=4})

dataukb$drink<-factor(dataukb$drink,levels = c(0,1,2,3,4),labels = c( "Never",
                                                                                                                    "Special occasions only" ,
                                                                                                                    "One to three times a month" ,
                                                                                                                    "1-4 times a week" ,
                                                                                                                    "Daily or almost daily" ))



level_k <-c("College or University degree" ="College or University degree","A levels/AS levels or equivalent"="Other"
                  ,"O levels/GCSEs or equivalent"="Other" ,"CSEs or equivalent"="Other" ,"NVQ or HND or HNC or equivalent"="Other"
                  ,"Other professional qualifications eg: nursing, teaching"="Other" )
dataukb$edu2<-recode_factor(dataukb$edu,!!!level_k)
dataukb$bmic[dataukb$bmic==99]=2
dataukb$bmic<-factor(dataukb$bmic,levels = c( 2,1,3,4),labels = c( "normal","lossweight","overweight","obesity" ))



levels(dataukb$eth)
dataukb$agecat<-cut(dataukb$age,breaks=c(35,55,65,75))
dataukb$agecat<-factor(dataukb$agecat,levels = c("(35,55]" ,"(55,65]" ,"(65,75]"),labels=c("1", "2","3"))




#diabete="all type diabetes according to UKB"
#insulin0 ="insulin at baseline"
```

```
#antdm0="any type of antidiabetic drugs use at baseline, excluding metformin and insulin use"
dm_s<-read.csv("D:/biobank/usedata/self_diabete.csv",header=TRUE, sep=",")    ###self-reported Diabetes
dataukb<-merge(dataukb,dm_s,by="n_eid")
#all type of type 2 diabete at baseline
dataukb$diabete_b=0
dataukb<-within(dataukb, {diabete_b[diabete==1&Survtime_diabete<=0]=1
diabete_b[insulin0=="Yes"|antdm0=="Yes"]=1
diabete_b[glucose>=11.1|HbA1c>=48]=1
diabete_b[dm_s=1]=1})

dataukb$HBP=0
dataukb<-within(dataukb,
                {HBP[hypert_s==1|high_bpD==1]=1
                HBP[bp_m==1]=1
                HBP[SBP>=140|DBP>=90]=1}) #hypert_s="self-reported hypertension"



# chol_m="Cholesterol lowering medication"
# chol_s="self-reported high cholesterol"
dataukb$chol_h<-0
dataukb<-within(dataukb,
                {chol_h[chol_s==1]=1
                chol_h[chol_m==1]=1})

##cancer before enrollment (cancer_b includes 33 missing value compared with bca)
dataukb$bca=0
dataukb<-within(dataukb, {bca[cancer==1 & survtime<=0]=1
bca[ca==1]=1})



##数据整理完毕---------------生成 dataukb-----------------

######----------绝经前后----------------- 1=已绝经；0=未绝经；99=unknown
dataukb$menopause1<-0
dataukb$menopause1[dataukb$menopause=="Yes"&dataukb$gender=="Female"]<-1
dataukb$menopause1[dataukb$gender=="Female"&dataukb$age>=53]<-1
dataukb$menopause1[dataukb$menopause=="No"&dataukb$gender=="Female"]<-0
dataukb$menopause1[is.na(dataukb$menopause1)&dataukb$gender=="Female"]<-99



dataukb<-within(dataukb,
                {breast_c_pos<-NA
                breast_c_pos[gender=="Female"&menopause1==1&breast_c=="Yes"]=1
                breast_c_pos[gender=="Female"&menopause1==1&breast_c=="No"]=0
                breast_c_pro<-NA
```

```
                          breast_c_pro[gender=="Female"&menopause1==0&breast_c=="Yes"]=1
                          breast_c_pro[gender=="Female"&menopause1==0&breast_c=="No"]=0})

dataukb$breast_c[dataukb$gender=="Male"]<-NA
dataukb$prostate_c[dataukb$gender=="Female"]<-NA
dataukb$Uterus_c[dataukb$gender=="Male"]<-NA
dataukb$ovary_c[dataukb$gender=="Male"]<-NA
dataukb$cervix_c[dataukb$gender=="Male"]<-NA



dataukb$oesophagitis_b<-ifelse(dataukb$oesophagitis==1&dataukb$Survtime_oesophagitis<=0,1,0)
dataukb$gerd_b<-ifelse(dataukb$GERD==1&dataukb$Survtime_GERD<=0,1,0) #/*gastro-oesophageal reflux (gord) / gastric reflux;*/
dataukb$ulcer_b<-ifelse((dataukb$Gu==1&dataukb$Survtime_Gu<=0) |(dataukb$Du==1&dataukb$Survtime_Du<=0),1,0)


######delete participants withdrew from the study (n=1298)------
# Survtime_L="Date lost to follow-up"
dataukb<-dataukb[is.na(dataukb$Survtime_L),]

#data_test<-dataukb[sample(nrow(dataukb),50000),]

#########----PPI_H2RA
## PPI_S0="any type of    PPI use at baseline"    h2ra_S0="any type of H2RA use at baseline"
dataukb<-within(dataukb,
                          {PPI_H2<-1
                          PPI_H2[h2ra_S0=="No"&PPI_S0=="No"]=1
                          PPI_H2[h2ra_S0=="No"&PPI_S0=="Yes"]=2
                          PPI_H2[h2ra_S0=="Yes"&PPI_S0=="No"]=3
                          PPI_H2[h2ra_S0=="Yes"&PPI_S0=="Yes"]=4})



dataukb$MI_b<-ifelse((dataukb$MI==1&dataukb$Survtime_MI<=0),1,0)
dataukb$CHD_b<-ifelse((dataukb$CHD==1&dataukb$Survtime_CHD<=0),1,0)
dataukb$CHF_b<-ifelse(((dataukb$CHF==1&dataukb$Survtime_CHF<=0)|dataukb$CHF_b==1),1,0)
dataukb$PVD_b<-ifelse(((dataukb$PVD==1&dataukb$Survtime_PVD<=0)),1,0)
dataukb$AF_b<-ifelse(((dataukb$AF==1&dataukb$Survtime_AF<=0)),1,0)
dataukb$stroke_b<-ifelse(((dataukb$stroke==1&dataukb$Survtime_stroke<=0)),1,0)
dataukb$UC_b<-ifelse(((dataukb$UC==1&dataukb$Survtime_UC<=0)),1,0)
dataukb$CD_b<-ifelse(((dataukb$CD==1&dataukb$Survtime_CD<=0)),1,0)
dataukb$IBD_b<-ifelse((dataukb$CD_b==1 | dataukb$UC_b==1),1,0)
dataukb$obstr_b<-ifelse(((dataukb$obstr==1&dataukb$Survtime_obstr<=0) |dataukb$obstr_b==1 ),1,0) #obstr=oesophageal stricture
dataukb$Ou_b<-ifelse(((dataukb$Ou==1&dataukb$Survtime_Ou<=0)),1,0) #/*oesophageal ulcer;*/
dataukb$Gu_b<-ifelse(((dataukb$Gu==1&dataukb$Survtime_Gu<=0)),1,0) #Gastric ulcer(GU)
dataukb$Du_b<-ifelse(((dataukb$Du==1&dataukb$Survtime_Du<=0)),1,0) #/*Duodenal ulcer*/
dataukb$Pu_b<-ifelse(((dataukb$Pu==1&dataukb$Survtime_Pu<=0)),1,0) #/*Peptic ulcer, site unspecified*/
dataukb$Gs_b<-ifelse(((dataukb$Gs==1&dataukb$Survtime_Gs<=0)),1,0) # Gastritis（GS）
dataukb$Dysp_b<-ifelse(((dataukb$Dysp==1&dataukb$Survtime_Dysp<=0)),1,0) #/*Disorders of function of stomach*/
```

```
dataukb$ugib_b<-ifelse(((dataukb$ugib==1&dataukb$Survtime_ugib<=0) |dataukb$ugib_b==1 ),1,0) #Upper gastrointestinal  bleeding and gastrointestinal  bleeding
dataukb$galst_b<-ifelse(((dataukb$galst==1&dataukb$Survtime_galst<=0)),1,0) # Cholelithiasis at baseline
dataukb$galcy_b<-ifelse(((dataukb$galcy==1&dataukb$Survtime_galcy<=0)),1,0) # Cholecystitis at baseline
dataukb$galag_b<-ifelse(((dataukb$galag==1&dataukb$Survtime_galag<=0)),1,0) # Cholangitis at baseline
dataukb$IBS_b<-ifelse(((dataukb$IBS==1&dataukb$Survtime_IBS<=0)),1,0) # IBS at baseline
dataukb$Survtime_IBD<-dataukb$Survtime_CD
dataukb$Survtime_IBD<-ifelse(dataukb$Survtime_IBD<dataukb$Survtime_UC,dataukb$Survtime_IBD,dataukb$Survtime_UC)
dataukb$gerd_b<-as.factor(dataukb$gerd_b)
dataukb$ulcer_b<-as.factor(dataukb$ulcer_b)
dataukb$COPD_b<-ifelse(((dataukb$COPD==1&dataukb$Survtime_COPD<=0)),1,0) # COPD at baseline
dataukb$asthma_b<-ifelse(((dataukb$asthma==1&dataukb$Survtime_asthma<=0)),1,0) # asthma at baseline
#bronchiectasis icd10:J47=1
dataukb$bronch_b<-ifelse(((dataukb$bronch==1&dataukb$Survtime_bronch<=0)),1,0) # bronch at baseline
#/* bronchiectasis */ icd10:J40=1 or J41=1  or J42=1  or J47=1
dataukb$bronchiectasis_b<-ifelse(((dataukb$bronchiectasis==1&dataukb$Survtime_bronchiectasis<=0)),1,0) # bronchiectasis at baseline
#"Dement","Survtime_Dement","AD","Survtime_AD","VD", "Survtime_VD","undemen", "Survtime_undemen",
dataukb$Dement_b<-ifelse(((dataukb$Dement==1&dataukb$Survtime_Dement<=0)),1,0) # Dement at baseline
dataukb$AD_b<-ifelse(((dataukb$AD==1&dataukb$Survtime_AD<=0)),1,0) # AD at baseline
dataukb$VD_b<-ifelse(((dataukb$VD==1&dataukb$Survtime_VD<=0)),1,0) # VD at baseline
dataukb$undemen_b<-ifelse(((dataukb$undemen==1&dataukb$Survtime_undemen<=0)),1,0) # undemen at baseline


dataukb$index<- ifelse((dataukb$oesophagitis_b==1)
                       | (dataukb$gerd_b==1)
                       | (dataukb$ulcer_b==1)
                       | (dataukb$Dysp==1),1,0)

dataukb$index<-factor(dataukb$index,levels = c(0,1),labels = c("No","Yes"))


dataukb<-within(dataukb,
             {gh=NA
             gh[Health_R=="Excellent"]=0
             gh[Health_R=="Good"]=1
             gh[Health_R=="Fair"]=2
             gh[Health_R=="Poor"]=3
             gh[Health_R=="Do not know"|Health_R=="Prefer not to answer"|Health_R==""]=4})
dataukb$Health_R<-dataukb$gh
dataukb$Health_R<-factor(dataukb$Health_R,levels = c(0,1,2,3,4),labels = c( "Excellent",
                                                                           "Good" ,
                                                                           "Fair" ,
                                                                           "Poor" ,
                                                                           "Do not know" ))


dataukb<-within(dataukb,
             {illness<-NA
             illness[illness_L=="No"]=0
```

```
                    illness[illness_L=="Yes"]=1
                    illness[illness_L=="Do not know"|illness_L=="Prefer not to answer"|illness_L==""]=2})
dataukb$illness_L<-dataukb$illness
dataukb$illness_L<-factor(dataukb$illness_L,levels = c(0,1,2),labels = c( "No",
                                                                             "Yes" ,
                                                                             "Do not know"    ))


#######data preparation----------
vars<-c("n_eid", "Survtime_CD", "Survtime_UC", "Survtime_IBS","survtime","UC" ,"CD","IBS","IBD","Survtime_IBD",
        "chol_s","hypert_s","db_s","DM", "high_bpD" , "diabete_b", "HBP","bca","chol_h",
        "UC_b",    "CD_b", "IBS_b","IBD_b",
        "oesophagitis_b",    "gerd_b", "obstr_b","Ou_b","Gu_b",    "Du_b", "Pu_b","Gs_b" ,"Dysp_b","ulcer_b", "ugib_b" ,
        "wc" ,"hip",    "height", "BMI","weight","SBP","gender","glucose","HbA1c",
        "centre","edu","age","eth","IDM","drinking","smoking","MET","Phy_act","fruit","vageta","F_V_t","fruit_g",
        "chol_m","bp_m","juejin","index",
        "lanso0",    "ome0", "panto0","rabe0","esome0","cime0",    "famo0", "niza0",
        "rani0", "insulin0" ,"metf0" ,"sulpho0" ,"Glita0","antdbt0" , "antdm0","simvas" ,"atovas", "statinO",
        "ACEI",    "ARBs",    "beteb", "Cach" , "TD" ,"LD","PSD", "antp","clogrel" , "antg",
        "PPI_S0","h2ra_S0",
        "statin","vitamin","mineral","ASP","Par","Ibu","NASIDS",
        "cancer_N", "noncancer_N","operations_N", "treatment_N",
        "bmic", "cen_obesity","obesity",
        "drinking4","age1","edu2","agecat","PPI_H2",
        "OCT", "HRT", "MetS_c", "MetS1", "Colorect_c", "colon_c","CRP","drink",
        "menopause1","Vitam_D","TG_H","HDL_L","bp_h","DM_H","DBP",
        "galltect","galst_b","galcy_b","galag_b","galcy_a","galcy_k",
        "diabete","Survtime_diabete", "BFP" , "WBFM",    "WBFFM" , "WBWM"    ,
        "BMI_I", "BMR",    "IWB",    "ILR",    "ILL",     "IAR",    "IAL",     "LFP_R",    "LFM_R",    "LFFM_R", "LPM_R", "LFP_L",    "LFM_L",
        "LFFM_L",    "LPM_L", "AFP_R", "AFM_R", "AFFM_R", "APM_R", "AFP_L", "AFM_L", "AFFM_L",     "APM_L",
        "TFP", "TFM", "TFFM" ,"PPI_H2","CRP","GDM",
        "COPD","Survtime_COPD","asthma","Survtime_asthma","bronchiectasis","Survtime_bronchiectasis","bronch","Survtime_bronch",
        "resdis","Survtime_resdis",
        "Dement","Survtime_Dement","AD","Survtime_AD","VD", "Survtime_VD","undemen", "Survtime_undemen",
        "death","Cancer_d","Survtime_d","lung_c","Source_IBS","Source_diabete",
        "galst","Survtime_galst","galcy","Survtime_galcy","galag","Survtime_galag",
        "Health_R","illness_L","CHOL","hp","LDL","HDL","TG","pancre_c","liver_c","rect_c"

)
data_IBS<-dataukb[vars]



###recode variables:missing rate <1%, the missing value was recoded into the largest groups
#########delete participants with IBS at baseline (n=25464)

data_IBS$BMI<- ifelse(is.na(data_IBS$BMI), 27.4, data_IBS$BMI)
data_IBS$SBP<- ifelse(is.na(data_IBS$SBP), 139.8, data_IBS$SBP)

data_IBS$smoking<- ifelse(data_IBS$smoking=="unkonwn/missing","3", data_IBS$smoking)
```

```
data_IBS$smoking<-factor(data_IBS$smoking,levels = c("1","2","3"),labels = c("Current" ,"Previous"     ,"Never" ))
table(data_IBS$smoking)

data_IBS$drinking4<- ifelse(data_IBS$drinking4=="unkonwn/missing","2", data_IBS$drinking4)
data_IBS$drinking4<-factor(data_IBS$drinking4,levels = c("1","2","3","4"),
                           labels = c("Daily or almost daily" ,"1-4 times a week" ,
                                      "One to three times a month" ,"Special occasions only/Never"     ))
table(data_IBS$drinking4)

table(data_IBS$fruit_g)
data_IBS$fruit_g<- ifelse(data_IBS$fruit_g=="Missing","1", data_IBS$fruit_g)
data_IBS$fruit_g<-factor(data_IBS$fruit_g,levels = c("1","2"),labels = c("No","Yes"))
table(data_IBS$fruit_g)

table(data_IBS$vitamin)
data_IBS$vitamin<- ifelse(data_IBS$vitamin=="Missing",1, data_IBS$vitamin)
data_IBS$vitamin<-factor(data_IBS$vitamin,levels = c(1,2),labels = c("No","Yes"))
table(data_IBS$vitamin)
data_IBS$NASIDS1<- ifelse(data_IBS$NASIDS==1 |data_IBS$Par==1,"Yes", "No")
table(data_IBS$NASIDS1)
table(data_IBS$mineral)
data_IBS$mineral<- ifelse(data_IBS$mineral=="Missing","1", data_IBS$mineral)
data_IBS$mineral<-factor(data_IBS$mineral,levels = c("1","2"),labels = c("No","Yes"))
table(data_IBS$mineral)

data_IBS$eth<- ifelse(data_IBS$eth=="unkonwn/missing","2", data_IBS$eth)
data_IBS$eth<-factor(data_IBS$eth,levels = c("1","2"),labels = c("Non-White","white"))
table(data_IBS$eth)

data_IBS$OCT<- ifelse((is.na(data_IBS$OCT) | data_IBS$OCT==99),"0", data_IBS$OCT)
data_IBS$OCT<-factor(data_IBS$OCT,levels = c("0","1"),labels = c("No","Yes"))
data_IBS$OCT<-ifelse(data_IBS$gender=="Male",NA,data_IBS$OCT)
data_IBS$OCT<-factor(data_IBS$OCT,levels = c("1","2"),labels = c("No","Yes"))
table(data_IBS$OCT)



data_IBS$menopause1<-ifelse(data_IBS$gender=="Male",NA,data_IBS$menopause1)
data_IBS$menopause1<-factor(data_IBS$menopause1,levels = c(0,1),labels = c("No","Yes"))
table(data_IBS$menopause1)

data_IBS$HRT<- ifelse((is.na(data_IBS$HRT) | data_IBS$HRT==99),0, data_IBS$HRT)
data_IBS$HRT<-ifelse(data_IBS$gender=="Male",NA,data_IBS$HRT)
data_IBS$HRT<-factor(data_IBS$HRT,levels = c("0","1"),labels = c("No","Yes"))
table(data_IBS$HRT)

data_IBS$ASP<-as.factor(data_IBS$ASP) # ASP="aspirin use"
data_IBS$Par<-as.factor(data_IBS$Par) # Par="paracetamol"
data_IBS$NASIDS<-as.factor(data_IBS$NASIDS) #NASIDS="non aspirin NASIDs use"
```

```
data_IBS$bca      <-as.factor(data_IBS$bca      ) # cancer at baseline
data_IBS$HBP<-as.factor(data_IBS$HBP)         #hypertention by doctor or self-reported or use antihypertensive drugs
data_IBS$diabete_b<-as.factor(data_IBS$diabete_b)         # any type of diabetes at baseline or use antidiabetic drugs or insulin at baseline
data_IBS$chol_h<-as.factor(data_IBS$chol_h) ##    self-reported high cholesterol or use Cholesterol lowering medication



data_IBS$antpl<-ifelse(data_IBS$antp=="Yes"|data_IBS$antg=="Yes","Yes", "No")    #antp="Antiplatelets" antg="Anticoagulants"
table(data_IBS$antpl)

data_IBS$age2=data_IBS$age1+data_IBS$Survtime_UC
data_IBS$MET_h=data_IBS$MET/60
data_IBS$age3=data_IBS$age1+data_IBS$Survtime_CD
data_IBS$age_ibd<-data_IBS$age1+data_IBS$Survtime_IBD
data_IBS$ppi_other<-ifelse(data_IBS$panto0=="Yes" | data_IBS$rabe0=="Yes","Yes", "No")
table(data_IBS$ppi_other)
data_IBS$non_ppi<-ifelse(data_IBS$ome0=="No"& data_IBS$ome0=="No"&data_IBS$lanso0=="No"&data_IBS$esome0=="No"&
data_IBS$ppi_other=="No","No","Yes")
data_IBS$gender1<-ifelse(dataukb$gender=="Male" |dataukb$gender=="Female",dataukb$gender,NA)
data_IBS$gender<-data_IBS$gender1
data_IBS$MET1<-data_IBS$MET/60

data_IBS$H2_other<-ifelse(data_IBS$cime0=="Yes" | data_IBS$famo0=="Yes" | data_IBS$niza0=="Yes","Yes", "No")
table(data_IBS$H2_other)
data_IBS$non_H2RA<-ifelse(data_IBS$cime0=="No"& data_IBS$famo0=="No"&data_IBS$niza0=="No"&data_IBS$rani0=="No","No","Yes")
data_IBS$liver_cb<-ifelse(((dataukb$liver_c==1&dataukb$survtime<=0)),1,0) # liver cancer at baseline
data_IBS$pancre_cb<-ifelse(((dataukb$pancre_c==1&dataukb$survtime<=0)),1,0) # pancrease cancer at baseline
#galst_b="Cholelithiasis at baseline"
#galcy_b="Cholecystitis at baseline"
#galag_b="Cholangitis at baseline"
data_IBS<-subset(data_IBS,data_IBS$galst_b==0&data_IBS$galcy_b==0&data_IBS$galag_b==0
                  &data_IBS$galltect==0&data_IBS$liver_cb==0&data_IBS$pancre_cb==0)
#######creat table1----------
tt<-table1(~ +age1+gender
           +eth +IDM+BMI
           +smoking+drink+MET1+fruit_g
           +HBP+chol_h+diabete_b
           +factor(oesophagitis_b)+factor(gerd_b)+factor(obstr_b)
           +factor(Ou_b)+factor(ulcer_b)+factor(Pu_b)+factor(Gs_b)
           +factor(Dysp_b)+factor(ulcer_b)+ factor(ugib_b)
           +vitamin+mineral
           +ASP+NASIDS+statin+ACEI+ARBs+beteb+h2ra_S0+Health_R+illness_L|PPI_S0,
           data=data_IBS, render.continuous=c(.="Mean (SD)"))



tt
write.table(tt,file = "D:\\中山七院工作\\文献\\PPI\\cholecystitis\\baseline1", sep = ",",
            col.names = NA,qmethod = "double")
```

```
tt_h2<-table1(~ +age1+gender
              +eth +IDM+BMI
              +smoking+drink+MET1+fruit_g
              +HBP+chol_h+diabete_b
              +factor(oesophagitis_b)+factor(gerd_b)+factor(obstr_b)
              +factor(Ou_b)+factor(ulcer_b)+factor(Pu_b)+factor(Gs_b)
              +factor(Dysp_b)+factor(ulcer_b)+ factor(ugib_b)
              +vitamin+mineral
              +ASP+NASIDS+statin+ACEI+ARBs+beteb+h2ra_S0+Health_R+illness_L|h2ra_S0,
              data=data_IBS, render.continuous=c(.="Mean (SD)"))


tt_h2
write.table(tt_h2,file = "D:\\中山七院工作\\文献\\PPI\\cholecystitis\\baseline_h2", sep = ",",
            col.names = NA,qmethod = "double")




##################PPI--Cholelithiasis associataion--------
###PPI and Cholelithiasis main analyses ------------------------

table(data_IBS[data_IBS$Survtime_galst>2,]$PPI_S0,
      data_IBS[data_IBS$Survtime_galst>2,]$galst)
aggregate(Survtime_galst~data_IBS[data_IBS$Survtime_galst>2,]$PPI_S0,
          data_IBS[data_IBS$Survtime_galst>2,],sum)


#Modle1
fit<- coxph(Surv(age1,Survtime_galst+age1,galst)~PPI_S0
            +strata(agecat)+strata(gender)+strata(centre)
            ,data=data_IBS[data_IBS$Survtime_galst>2,])
HR <- round(exp(coef(fit)), 2)
CI <- round(exp(confint(fit)), 2)
P <- round(coef(summary(fit))[,5], 3)
colnames(CI) <- c("Lower", "Higher")
out<- as.data.frame(cbind(HR, CI, P))
out1<- out[1,]
out1



#Modle2
fit<- coxph(Surv(age1,Survtime_galst+age1,galst)~PPI_S0
            +strata(agecat)+strata(gender)+strata(centre)
            +eth +IDM+bmic
            +smoking+drinking4+Phy_act+fruit_g
            +HBP+chol_h+diabete_b+index
            +Health_R+illness_L
```

```
              ,data=data_IBS[data_IBS$Survtime_galst>2,])
HR <- round(exp(coef(fit)), 2)
CI <- round(exp(confint(fit)), 2)
P <- round(coef(summary(fit))[,5], 3)
colnames(CI) <- c("Lower", "Higher")
out<- as.data.frame(cbind(HR, CI, P))
out2<- out[1,]
out2


#Modle3
# centre   =data generated from different centre
fit<- coxph(Surv(age1,Survtime_galst+age1,galst)~PPI_S0
              +strata(agecat)+strata(gender)+strata(centre)
              +eth +IDM+bmic
              +smoking+drinking4+Phy_act+fruit_g
              +HBP+chol_h+diabete_b+index
              +Health_R+illness_L
              +ASP+NASIDS+statin+ACEI+ARBs+beteb+vitamin+mineral
              ,data=data_IBS[data_IBS$Survtime_galst>2,])
HR <- round(exp(coef(fit)), 2)
CI <- round(exp(confint(fit)), 2)
P <- round(coef(summary(fit))[,5], 3)
colnames(CI) <- c("Lower", "Higher")
out<- as.data.frame(cbind(HR, CI, P))
out3<- out[1,]
out3



out_IBS<- as.data.frame(rbind(out1,out2,out3))
out_IBS
write.table(out_IBS, file = "D:\\中山七院工作\\文献\\PPI\\cholecystitis\\all_ppi.csv", sep = ",",
              col.names = NA,qmethod = "double")

#Colorect_c", "colon_c"

fit<- coxph(Surv(age1,survtime+age1,Colorect_c)~PPI_S0
              +strata(agecat)+strata(gender)+strata(centre)
              +eth +IDM+bmic
              +smoking+drinking4+Phy_act+fruit_g
              +HBP+chol_h+diabete_b+index
              +Health_R+illness_L
              +ASP+NASIDS+statin+ACEI+ARBs+beteb+vitamin+mineral
              ,data=data_IBS[data_IBS$survtime>2,])
HR <- round(exp(coef(fit)), 2)
CI <- round(exp(confint(fit)), 2)
P <- round(coef(summary(fit))[,5], 3)
colnames(CI) <- c("Lower", "Higher")
```

```
out<- as.data.frame(cbind(HR, CI, P))
out3<- out[1,]
out3


fit<- coxph(Surv(age1,survtime+age1,colon_c)~PPI_S0
            +strata(agecat)+strata(gender)+strata(centre)
            +eth +IDM+bmic
            +smoking+drinking4+Phy_act+fruit_g
            +HBP+chol_h+diabete_b+index
            +Health_R+illness_L
            +ASP+NASIDS+statin+ACEI+ARBs+beteb+vitamin+mineral
            ,data=data_IBS[data_IBS$survtime>2,])
HR <- round(exp(coef(fit)), 2)
CI <- round(exp(confint(fit)), 2)
P <- round(coef(summary(fit))[,5], 3)
colnames(CI) <- c("Lower", "Higher")
out<- as.data.frame(cbind(HR, CI, P))
out3<- out[1,]
out3


fit<- coxph(Surv(age1,survtime+age1,rect_c)~PPI_S0
            +strata(agecat)+strata(gender)+strata(centre)
            +eth +IDM+bmic
            +smoking+drinking4+Phy_act+fruit_g
            +HBP+chol_h+diabete_b+index
            +Health_R+illness_L
            +ASP+NASIDS+statin+ACEI+ARBs+beteb+vitamin+mineral
            ,data=data_IBS[data_IBS$survtime>2,])
HR <- round(exp(coef(fit)), 2)
CI <- round(exp(confint(fit)), 2)
P <- round(coef(summary(fit))[,5], 3)
colnames(CI) <- c("Lower", "Higher")
out<- as.data.frame(cbind(HR, CI, P))
out3<- out[1,]
out3


fit<- coxph(Surv(age1,Survtime_galst+age1,galst)~PPI_S0
            +strata(agecat)+strata(gender)+strata(centre)
            +eth +IDM+bmic
            +smoking+drinking4+Phy_act+fruit_g
            +HBP+chol_h+diabete_b+index
            +Health_R+illness_L
            +ASP+NASIDS+statin+ACEI+ARBs+beteb+vitamin+mineral
            ,data=data_IBS[data_IBS$Survtime_galst>2,])
```

```
library(MatchIt)
vars2<-c("n_eid", "PPI_S0","h2ra_S0","age1","gender" ,"IDM","bmic","eth","centre",
          "smoking","drinking4","Phy_act","fruit_g","index",
          "HBP","chol_h","diabete_b","Health_R","illness_L",
          "ASP","NASIDS","vitamin","mineral","Survtime_galst","galst","agecat",
          "statin","ACEI","ARBs","beteb","rect_c","colon_c","Colorect_c"
)
data_PS<-data_IBS[vars2]
summary(data_PS)
data_PS<-data_PS[is.na(data_PS$agecat)=="FALSE",]
data_PS<-data_PS[is.na(data_PS$IDM)=="FALSE",] #######必须要删除空缺值
summary(data_PS)
set.seed(2020)



log1<-glm(formula = PPI_S0~agecat+gender+strata(centre)
           +eth +IDM+bmic
           +smoking+drinking4+Phy_act+fruit_g
           +HBP+chol_h+diabete_b+index
           +Health_R+illness_L
           +ASP+NASIDS+statin+ACEI+ARBs+beteb+vitamin+mineral,family = binomial(),
           data=data_PS)
PS<-fitted(log1)
SD(logit(PS))
0.2*SD(logit(PS))      ###这个即为卡钳值  caliper


psmmatch1=matchit(PPI_S0~agecat+gender+strata(centre)
                  +eth +IDM+bmic
                  +smoking+drinking4+Phy_act+fruit_g
                  +HBP+chol_h+diabete_b+index
                  +Health_R+illness_L
                  +ASP+NASIDS+statin+ACEI+ARBs+beteb+vitamin+mineral, method="nearest",ratio=4,caliper=0.2706794,
                  data=data_PS)      ####进行 PS 匹配

summary(psmmatch1)    ###可以计算 PS 匹配后每个变量的标准化差异

matchdata1=match.data(psmmatch1) ##得到匹配之后的数据集（可用于后续分析）
summary(matchdata1)

vars3<-c("n_eid", "survtime")
data_PS1<-data_IBS[vars3]
matchdata2<-merge(matchdata1,data_PS1,by="n_eid")

fit<- coxph(Surv(age1,age1+survtime,colon_c)~PPI_S0
```

```
                  ,data=matchdata2[matchdata2$survtime>2,])
HR <- round(exp(coef(fit)), 2)
CI <- round(exp(confint(fit)), 2)
P <- round(coef(summary(fit))[,5], 3)
colnames(CI) <- c("Lower", "Higher")
out<- as.data.frame(cbind(HR, CI, P))
out1<- out[1,]
out1

fit<- coxph(Surv(age1,age1+survtime,Colorect_c)~PPI_S0
                  ,data=matchdata2[matchdata2$survtime>2,])
HR <- round(exp(coef(fit)), 2)
CI <- round(exp(confint(fit)), 2)
P <- round(coef(summary(fit))[,5], 3)
colnames(CI) <- c("Lower", "Higher")
out<- as.data.frame(cbind(HR, CI, P))
out2<- out[1,]
out2

fit<- coxph(Surv(age1,age1+survtime,rect_c)~PPI_S0
                  ,data=matchdata2[matchdata2$survtime>2,])
HR <- round(exp(coef(fit)), 2)
CI <- round(exp(confint(fit)), 2)
P <- round(coef(summary(fit))[,5], 3)
colnames(CI) <- c("Lower", "Higher")
out<- as.data.frame(cbind(HR, CI, P))
out3<- out[1,]
out3

ppi_IBD<- as.data.frame(rbind(out1, out2,       out3))
ppi_IBD

#for each type of PPI and galst------------
ppi1<-table(data_IBS[data_IBS$Survtime_galst>2&data_IBS$non_ppi=="No",]$galst)
ppi2<-aggregate(Survtime_galst~data_IBS[data_IBS$Survtime_galst>2,]$non_ppi,
                        data_IBS[data_IBS$Survtime_galst>2,],sum)
out0<- as.data.frame(cbind(ppi1,ppi2))
out0

ome01<-table(data_IBS[data_IBS$Survtime_galst>2,]$ome0,
                   data_IBS[data_IBS$Survtime_galst>2,]$galst)
ome02<-aggregate(Survtime_galst~data_IBS[data_IBS$Survtime_galst>2,]$ome0,
                         data_IBS[data_IBS$Survtime_galst>2,],sum)
out1<- as.data.frame(cbind(ome01,ome02))
out1


lanso1<-table(data_IBS[data_IBS$Survtime_galst>2,]$lanso0,
                    data_IBS[data_IBS$Survtime_galst>2,]$galst)
```

```
lanso2<-aggregate(Survtime_galst~data_IBS[data_IBS$Survtime_galst>2,]$lanso0,
                  data_IBS[data_IBS$Survtime_galst>2,],sum)
out2<- as.data.frame(cbind(lanso1,lanso2))
out2


esome1<-table(data_IBS[data_IBS$Survtime_galst>2,]$esome0,
              data_IBS[data_IBS$Survtime_galst>2,]$galst)
esome2<-aggregate(Survtime_galst~data_IBS[data_IBS$Survtime_galst>2,]$esome0,
                  data_IBS[data_IBS$Survtime_galst>2,],sum)
out3<- as.data.frame(cbind(esome1,esome2))
out3


ppi_other1<-table(data_IBS[data_IBS$Survtime_galst>2,]$ppi_other,
                  data_IBS[data_IBS$Survtime_galst>2,]$galst)
ppi_other2<-aggregate(Survtime_galst~data_IBS[data_IBS$Survtime_galst>2,]$ppi_other,
                      data_IBS[data_IBS$Survtime_galst>2,],sum)
out4<- as.data.frame(cbind(ppi_other1,ppi_other2))
out4

ppi_years_IBD<- as.data.frame(cbind(out0, out1, out2,     out3,          out4))
ppi_years_IBD
write.table(ppi_years_IBD, file = "D:\\中山七院工作\\文献\\PPI\\cholecystitis\\eachPPI_years.csv", sep = ",",
            col.names = NA,qmethod = "double")
##multivar-adjusted

# ome0 ="omeprazole at baseline"
fit<- coxph(Surv(age1,Survtime_galst+age1,galst)~ome0
            +strata(agecat)+strata(gender)+strata(centre)
            +eth +IDM+bmic
            +smoking+drinking4+Phy_act+fruit_g
            +HBP+chol_h+diabete_b+index
            +ASP+NASIDS+statin+ACEI+ARBs+beteb+vitamin+mineral+Health_R+illness_L
            ,data=data_IBS[data_IBS$Survtime_galst>2&(data_IBS$non_ppi=="No"|data_IBS$ome0=="Yes"),])
HR <- round(exp(coef(fit)), 2)
CI <- round(exp(confint(fit)), 2)
P <- round(coef(summary(fit))[,5], 3)
colnames(CI) <- c("Lower", "Higher")
out<- as.data.frame(cbind(HR, CI, P))
out_om<- out[1,]
out_om

# "lansoprazole at baseline"
fit<- coxph(Surv(age1,Survtime_galst+age1,galst)~lanso0
            +strata(agecat)+strata(gender)+strata(centre)
            +eth +IDM+bmic
            +smoking+drinking4+Phy_act+fruit_g
            +HBP+chol_h+diabete_b+index
```

```
                    +ASP+NASIDS+statin+ACEI+ARBs+beteb+vitamin+mineral+Health_R+illness_L
                    ,data=data_IBS[data_IBS$Survtime_galst>2&(data_IBS$non_ppi=="No"|data_IBS$lanso0=="Yes"),])
HR <- round(exp(coef(fit)), 2)
CI <- round(exp(confint(fit)), 2)
P <- round(coef(summary(fit))[,5], 3)
colnames(CI) <- c("Lower", "Higher")
out<- as.data.frame(cbind(HR, CI, P))
out_l<- out[1,]
out_l


# esome0="esomeprazole at baseline"
fit<- coxph(Surv(age1,Survtime_galst+age1,galst)~esome0
                    +strata(agecat)+strata(gender)+strata(centre)
                    +eth +IDM+bmic
                    +smoking+drinking4+Phy_act+fruit_g
                    +HBP+chol_h+diabete_b+index
                    +ASP+NASIDS+statin+ACEI+ARBs+beteb+vitamin+mineral+Health_R+illness_L
                    ,data=data_IBS[data_IBS$Survtime_galst>2&(data_IBS$non_ppi=="No"|data_IBS$esome0=="Yes"),])
HR <- round(exp(coef(fit)), 2)
CI <- round(exp(confint(fit)), 2)
P <- round(coef(summary(fit))[,5], 3)
colnames(CI) <- c("Lower", "Higher")
out<- as.data.frame(cbind(HR, CI, P))
out_es<- out[1,]
out_es


# ppi_other="other ppis at baseline"
fit<- coxph(Surv(age1,Survtime_galst+age1,galst)~ppi_other
                    +strata(agecat)+strata(gender)+strata(centre)
                    +eth +IDM+bmic
                    +smoking+drinking4+Phy_act+fruit_g
                    +HBP+chol_h+diabete_b+index
                    +ASP+NASIDS+statin+ACEI+ARBs+beteb+vitamin+mineral+Health_R+illness_L
                    ,data=data_IBS[data_IBS$Survtime_galst>2&(data_IBS$non_ppi=="No"|data_IBS$ppi_other=="Yes"),])
HR <- round(exp(coef(fit)), 2)
CI <- round(exp(confint(fit)), 2)
P <- round(coef(summary(fit))[,5], 3)
colnames(CI) <- c("Lower", "Higher")
out<- as.data.frame(cbind(HR, CI, P))
out_other<- out[1,]
out_other


out_ppis<- as.data.frame(rbind(out_om,out_l,out_es,out_other))
out_ppis
write.table(out_ppis, file = "D:\\中山七院工作\\文献\\PPI\\cholecystitis\\eachppi_galst.csv", sep = ",",
                    col.names = NA,qmethod = "double")
```

```
########subgroup analysis for galst --PPI--------

data_IBS$bmi_fen<-1
data_IBS$bmi_fen[data_IBS$bmic!="obesity"]=0
data_IBS$bmi_fen<-factor(data_IBS$bmi_fen,levels = c(0,1),labels = c( "<30",">=30" ))


data_IBS$age_fen<-0
data_IBS$age_fen[data_IBS$age1 >=60]=1
data_IBS$age_fen<-factor(data_IBS$age_fen,levels = c(0,1),labels = c( "<60",">=60" ))


data_IBS$smk_fen<-0
data_IBS$smk_fen[data_IBS$smoking!="Never"]=1
data_IBS$smk_fen<-factor(data_IBS$smk_fen,levels = c(0,1),labels = c( "No smoker","Smoker" ))

data_IBS$drk_fen<-1
data_IBS$drk_fen[data_IBS$drinking=="Never"|data_IBS$drinking=="unkonwn/missing"]=0
data_IBS$drk_fen<-factor(data_IBS$drk_fen,levels = c(0,1),labels = c( "No drinker","Drinker" ))


data_IBS$act_fen<-0
data_IBS$act_fen[data_IBS$MET >=1771]=1
data_IBS$act_fen<-factor(data_IBS$act_fen,levels = c(0,1),labels = c( "activity < median","activity >= median" ))


#####For Male
fit<- coxph(Surv(age1,Survtime_galst+age1,galst)~PPI_S0
            +strata(agecat)+strata(centre)
            +eth +IDM+bmic
            +smoking+drinking4+Phy_act+fruit_g
            +HBP+chol_h+diabete_b+index
            +ASP+NASIDS+statin+ACEI+ARBs+beteb+vitamin+mineral+Health_R+illness_L
            ,data=data_IBS[data_IBS$Survtime_galst>2&data_IBS$gender=="Male",])
HR <- round(exp(coef(fit)), 2)
CI <- round(exp(confint(fit)), 2)
P <- round(coef(summary(fit))[,5], 3)
colnames(CI) <- c("Lower", "Higher")
out<- as.data.frame(cbind(HR, CI, P))
out_male<- out[1,]
out_male

#####For Female
```

```
fit<- coxph(Surv(age1,Survtime_galst+age1,galst)~PPI_S0
            +strata(agecat)+strata(centre)
            +eth +IDM+bmic
            +smoking+drinking4+Phy_act+fruit_g
            +HBP+chol_h+diabete_b+index
            +ASP+NASIDS+statin+ACEI+ARBs+beteb+vitamin+mineral+Health_R+illness_L
            +menopause1+HRT+OCT
            ,data=data_IBS[data_IBS$Survtime_galst>2&data_IBS$gender=="Female",])
HR <- round(exp(coef(fit)), 2)
CI <- round(exp(confint(fit)), 2)
P <- round(coef(summary(fit))[,5], 3)
colnames(CI) <- c("Lower", "Higher")
out<- as.data.frame(cbind(HR, CI, P))
out_female<- out[1,]
out_female


#bmi_fen
fit<- coxph(Surv(age1,Survtime_galst+age1,galst)~PPI_S0
            +strata(agecat)+strata(gender)+strata(centre)
            +eth +IDM+BMI
            +smoking+drinking4+Phy_act+fruit_g
            +HBP+chol_h+diabete_b+index
            +ASP+NASIDS+statin+ACEI+ARBs+beteb+vitamin+mineral+Health_R+illness_L
            ,data=data_IBS[data_IBS$Survtime_galst>2&data_IBS$bmi_fen=="<30",])
HR <- round(exp(coef(fit)), 2)
CI <- round(exp(confint(fit)), 2)
P <- round(coef(summary(fit))[,5], 3)
colnames(CI) <- c("Lower", "Higher")
out<- as.data.frame(cbind(HR, CI, P))
bmi_out0<- out[1,]
bmi_out0


fit<- coxph(Surv(age1,Survtime_galst+age1,galst)~PPI_S0
            +strata(agecat)+strata(gender)+strata(centre)
            +eth +IDM+BMI
            +smoking+drinking4+Phy_act+fruit_g
            +HBP+chol_h+diabete_b+index
            +ASP+NASIDS+statin+ACEI+ARBs+beteb+vitamin+mineral+Health_R+illness_L
            ,data=data_IBS[data_IBS$Survtime_galst>2&data_IBS$bmi_fen==">=30",])
HR <- round(exp(coef(fit)), 2)
CI <- round(exp(confint(fit)), 2)
P <- round(coef(summary(fit))[,5], 3)
colnames(CI) <- c("Lower", "Higher")
out<- as.data.frame(cbind(HR, CI, P))
bmi_out1<- out[1,]
bmi_out1
```

```
#age
fit<- coxph(Surv(age1,Survtime_galst+age1,galst)~PPI_S0
            +strata(agecat)+strata(gender)+strata(centre)
            +eth +IDM+bmic
            +smoking+drinking4+Phy_act+fruit_g
            +HBP+chol_h+diabete_b+index
            +ASP+NASIDS+statin+ACEI+ARBs+beteb+vitamin+mineral+Health_R+illness_L
            ,data=data_IBS[data_IBS$Survtime_galst>2&data_IBS$age_fen=="<60",])
HR <- round(exp(coef(fit)), 2)
CI <- round(exp(confint(fit)), 2)
P <- round(coef(summary(fit))[,5], 3)
colnames(CI) <- c("Lower", "Higher")
out<- as.data.frame(cbind(HR, CI, P))
age_out0<- out[1,]
age_out0


fit<- coxph(Surv(age1,Survtime_galst+age1,galst)~PPI_S0
            +strata(agecat)+strata(gender)+strata(centre)
            +eth +IDM+bmic
            +smoking+drinking4+Phy_act+fruit_g
            +HBP+chol_h+diabete_b+index
            +ASP+NASIDS+statin+ACEI+ARBs+beteb+vitamin+mineral+Health_R+illness_L
            ,data=data_IBS[data_IBS$Survtime_galst>2&data_IBS$age_fen==">=60",])
HR <- round(exp(coef(fit)), 2)
CI <- round(exp(confint(fit)), 2)
P <- round(coef(summary(fit))[,5], 3)
colnames(CI) <- c("Lower", "Higher")
out<- as.data.frame(cbind(HR, CI, P))
age_out1<- out[1,]
age_out1




#activity

fit<- coxph(Surv(age1,Survtime_galst+age1,galst)~PPI_S0
            +strata(agecat)+strata(gender)+strata(centre)
            +eth +IDM+bmic
            +smoking+drinking4+fruit_g
            +HBP+chol_h+diabete_b+index
            +ASP+NASIDS+statin+ACEI+ARBs+beteb+vitamin+mineral+Health_R+illness_L
            ,data=data_IBS[data_IBS$Survtime_galst>2&data_IBS$act_fen=="activity < median",])
HR <- round(exp(coef(fit)), 2)
CI <- round(exp(confint(fit)), 2)
P <- round(coef(summary(fit))[,5], 3)
colnames(CI) <- c("Lower", "Higher")
```

```
out<- as.data.frame(cbind(HR, CI, P))
act_out0<- out[1,]
act_out0


fit<- coxph(Surv(age1,Survtime_galst+age1,galst)~PPI_S0
            +strata(agecat)+strata(gender)+strata(centre)
            +eth +IDM+bmic
            +smoking+drinking4+fruit_g
            +HBP+chol_h+diabete_b+index
            +ASP+NASIDS+statin+ACEI+ARBs+beteb+vitamin+mineral+Health_R+illness_L
            ,data=data_IBS[data_IBS$Survtime_galst>2&data_IBS$act_fen=="activity >= median",])
HR <- round(exp(coef(fit)), 2)
CI <- round(exp(confint(fit)), 2)
P <- round(coef(summary(fit))[,5], 3)
colnames(CI) <- c("Lower", "Higher")
out<- as.data.frame(cbind(HR, CI, P))
act_out1<- out[1,]
act_out1


##smoking
fit<- coxph(Surv(age1,Survtime_galst+age1,galst)~PPI_S0
            +strata(agecat)+strata(gender)+strata(centre)
            +eth +IDM+bmic
            +drinking4+Phy_act+fruit_g
            +HBP+chol_h+diabete_b+index
            +ASP+NASIDS+statin+ACEI+ARBs+beteb+vitamin+mineral+Health_R+illness_L
            ,data=data_IBS[data_IBS$Survtime_galst>2&data_IBS$smk_fen=="No smoker",])
HR <- round(exp(coef(fit)), 2)
CI <- round(exp(confint(fit)), 2)
P <- round(coef(summary(fit))[,5], 3)
colnames(CI) <- c("Lower", "Higher")
out<- as.data.frame(cbind(HR, CI, P))
smk_out0<- out[1,]
smk_out0


fit<- coxph(Surv(age1,Survtime_galst+age1,galst)~PPI_S0
            +strata(agecat)+strata(gender)+strata(centre)
            +eth +IDM+bmic
            +drinking4+Phy_act+fruit_g
            +HBP+chol_h+diabete_b+index
            +ASP+NASIDS+statin+ACEI+ARBs+beteb+vitamin+mineral+Health_R+illness_L
            ,data=data_IBS[data_IBS$Survtime_galst>2&data_IBS$smk_fen=="Smoker",])
HR <- round(exp(coef(fit)), 2)
CI <- round(exp(confint(fit)), 2)
P <- round(coef(summary(fit))[,5], 3)
colnames(CI) <- c("Lower", "Higher")
```

```
out<- as.data.frame(cbind(HR, CI, P))
smk_out1<- out[1,]
smk_out1




#drinking
fit<- coxph(Surv(age1,Survtime_galst+age1,galst)~PPI_S0
            +strata(agecat)+strata(gender)+strata(centre)
            +eth +IDM+bmic
            +smoking+Phy_act+fruit_g
            +HBP+chol_h+diabete_b+index
            +ASP+NASIDS+statin+ACEI+ARBs+beteb+vitamin+mineral+Health_R+illness_L
            ,data=data_IBS[data_IBS$Survtime_galst>2&data_IBS$drk_fen=="No drinker",])
HR <- round(exp(coef(fit)), 2)
CI <- round(exp(confint(fit)), 2)
P <- round(coef(summary(fit))[,5], 3)
colnames(CI) <- c("Lower", "Higher")
out<- as.data.frame(cbind(HR, CI, P))
drk_out0<- out[1,]
drk_out0


fit<- coxph(Surv(age1,Survtime_galst+age1,galst)~PPI_S0
            +strata(agecat)+strata(gender)+strata(centre)
            +eth +IDM+bmic
            +smoking+Phy_act+fruit_g
            +HBP+chol_h+diabete_b+index
            +ASP+NASIDS+statin+ACEI+ARBs+beteb+vitamin+mineral+Health_R+illness_L
            ,data=data_IBS[data_IBS$Survtime_galst>2&data_IBS$drk_fen=="Drinker",])
HR <- round(exp(coef(fit)), 2)
CI <- round(exp(confint(fit)), 2)
P <- round(coef(summary(fit))[,5], 3)
colnames(CI) <- c("Lower", "Higher")
out<- as.data.frame(cbind(HR, CI, P))
drk_out1<- out[1,]
drk_out1




#fruit and vegetable
fit<- coxph(Surv(age1,Survtime_galst+age1,galst)~PPI_S0
            +strata(agecat)+strata(gender)+strata(centre)
            +eth +IDM+bmic
            +smoking+drinking4+Phy_act
            +HBP+chol_h+diabete_b+index
            +ASP+NASIDS+statin+ACEI+ARBs+beteb+vitamin+mineral+Health_R+illness_L
            ,data=data_IBS[data_IBS$Survtime_galst>2&data_IBS$fruit_g=="No",])
```

```
HR <- round(exp(coef(fit)), 2)
CI <- round(exp(confint(fit)), 2)
P <- round(coef(summary(fit))[,5], 3)
colnames(CI) <- c("Lower", "Higher")
out<- as.data.frame(cbind(HR, CI, P))
out_fru0<- out[1,]
out_fru0


fit<- coxph(Surv(age1,Survtime_galst+age1,galst)~PPI_S0
            +strata(agecat)+strata(gender)+strata(centre)
            +eth +IDM+bmic
            +smoking+drinking4+Phy_act
            +HBP+chol_h+diabete_b+index
            +ASP+NASIDS+statin+ACEI+ARBs+beteb+vitamin+mineral+Health_R+illness_L
            ,data=data_IBS[data_IBS$Survtime_galst>2&data_IBS$fruit_g=="Yes",])
HR <- round(exp(coef(fit)), 2)
CI <- round(exp(confint(fit)), 2)
P <- round(coef(summary(fit))[,5], 3)
colnames(CI) <- c("Lower", "Higher")
out<- as.data.frame(cbind(HR, CI, P))
out_fru1<- out[1,]
out_fru1



#NASIDS
fit<- coxph(Surv(age1,Survtime_galst+age1,galst)~PPI_S0
            +strata(agecat)+strata(gender)+strata(centre)
            +eth +IDM+bmic
            +smoking+drinking4+Phy_act+fruit_g
            +HBP+chol_h+diabete_b+index
            +ASP+statin+ACEI+ARBs+beteb+vitamin+mineral+Health_R+illness_L
            ,data=data_IBS[data_IBS$Survtime_galst>2&data_IBS$NASIDS==0,])
HR <- round(exp(coef(fit)), 2)
CI <- round(exp(confint(fit)), 2)
P <- round(coef(summary(fit))[,5], 3)
colnames(CI) <- c("Lower", "Higher")
out<- as.data.frame(cbind(HR, CI, P))
NASIDS_out0<- out[1,]
NASIDS_out0


fit<- coxph(Surv(age1,Survtime_galst+age1,galst)~PPI_S0
            +strata(agecat)+strata(gender)+strata(centre)
            +eth +IDM+bmic
            +smoking+drinking4+Phy_act+fruit_g
            +HBP+chol_h+diabete_b+index
            +ASP+statin+ACEI+ARBs+beteb+vitamin+mineral+Health_R+illness_L
```

```
            ,data=data_IBS[data_IBS$Survtime_galst>2&data_IBS$NASIDS==1,])
HR <- round(exp(coef(fit)), 2)
CI <- round(exp(confint(fit)), 2)
P <- round(coef(summary(fit))[,5], 3)
colnames(CI) <- c("Lower", "Higher")
out<- as.data.frame(cbind(HR, CI, P))
NASIDS_out1<- out[1,]
NASIDS_out1


#ASP
fit<- coxph(Surv(age1,Survtime_galst+age1,galst)~PPI_S0
            +strata(agecat)+strata(gender)+strata(centre)
            +eth +IDM+bmic
            +smoking+drinking4+Phy_act+fruit_g
            +HBP+chol_h+diabete_b+index
            +NASIDS+statin+ACEI+ARBs+beteb+vitamin+mineral+Health_R+illness_L
            ,data=data_IBS[data_IBS$Survtime_galst>2&data_IBS$ASP==0,])
HR <- round(exp(coef(fit)), 2)
CI <- round(exp(confint(fit)), 2)
P <- round(coef(summary(fit))[,5], 3)
colnames(CI) <- c("Lower", "Higher")
out<- as.data.frame(cbind(HR, CI, P))
ASP_out0<- out[1,]
ASP_out0


fit<- coxph(Surv(age1,Survtime_galst+age1,galst)~PPI_S0
            +strata(agecat)+strata(gender)+strata(centre)
            +eth +IDM+bmic
            +smoking+drinking4+Phy_act+fruit_g
            +HBP+chol_h+diabete_b+index
            +NASIDS+statin+ACEI+ARBs+beteb+vitamin+mineral+Health_R+illness_L
            ,data=data_IBS[data_IBS$Survtime_galst>2&data_IBS$ASP==1,])
HR <- round(exp(coef(fit)), 2)
CI <- round(exp(confint(fit)), 2)
P <- round(coef(summary(fit))[,5], 3)
colnames(CI) <- c("Lower", "Higher")
out<- as.data.frame(cbind(HR, CI, P))
ASP_out1<- out[1,]
ASP_out1



#GERD
fit<- coxph(Surv(age1,Survtime_galst+age1,galst)~PPI_S0
            +strata(agecat)+strata(gender)+strata(centre)
            +eth +IDM+bmic
```

```
                    +smoking+drinking4+Phy_act+fruit_g
                    +HBP+chol_h+diabete_b
                    +ASP+NASIDS+statin+ACEI+ARBs+beteb+vitamin+mineral+Health_R+illness_L
                    ,data=data_IBS[data_IBS$Survtime_galst>2&data_IBS$gerd_b==0,])
HR <- round(exp(coef(fit)), 2)
CI <- round(exp(confint(fit)), 2)
P <- round(coef(summary(fit))[,5], 3)
colnames(CI) <- c("Lower", "Higher")
out<- as.data.frame(cbind(HR, CI, P))
gerd_out0<- out[1,]
gerd_out0


fit<- coxph(Surv(age1,Survtime_galst+age1,galst)~PPI_S0
                    +strata(agecat)+strata(gender)+strata(centre)
                    +eth +IDM+bmic
                    +smoking+drinking4+Phy_act+fruit_g
                    +HBP+chol_h+diabete_b
                    +ASP+NASIDS+statin+ACEI+ARBs+beteb+vitamin+mineral+Health_R+illness_L
                    ,data=data_IBS[data_IBS$Survtime_galst>2&data_IBS$gerd_b==1,])
HR <- round(exp(coef(fit)), 2)
CI <- round(exp(confint(fit)), 2)
P <- round(coef(summary(fit))[,5], 3)
colnames(CI) <- c("Lower", "Higher")
out<- as.data.frame(cbind(HR, CI, P))
gerd_out1<- out[1,]
gerd_out1

subgroup_IBD<- as.data.frame(rbind(out_male,out_female,age_out0,age_out1,bmi_out0,bmi_out1,
                                                       smk_out0,smk_out1,drk_out0,drk_out1,
                                                       act_out0,act_out1,out_fru0,out_fru1,
                                                       NASIDS_out0,NASIDS_out1,ASP_out0,ASP_out1,
                                                       gerd_out0,gerd_out1))
subgroup_IBD

write.table(subgroup_IBD, file = "D:\\中山七院工作\\文献\\PPI\\cholecystitis\\subgroup_ppi.csv", sep = ",",
                    col.names = NA,qmethod = "double")



### p interaction for ppi--galst association--------

#gender
fit<- coxph(Surv(age1,Survtime_galst+age1,galst)~PPI_S0*gender
                    +strata(agecat)+strata(centre)
                    +eth +IDM+bmic
                    +smoking+drinking4+Phy_act+fruit_g
                    +HBP+chol_h+diabete_b+index
                    +ASP+NASIDS+statin+ACEI+ARBs+beteb+vitamin+mineral+Health_R+illness_L
```

```
                ,data=data_IBS[data_IBS$Survtime_galst>2,])
HR <- round(exp(coef(fit)), 2)
CI <- round(exp(confint(fit)), 2)
P <- round(coef(summary(fit))[,5], 3)
colnames(CI) <- c("Lower", "Higher")
out0<- as.data.frame(cbind(HR, CI, P))
out0



#bmi_fen
fit<- coxph(Surv(age1,Survtime_galst+age1,galst)~PPI_S0*bmi_fen
                +strata(agecat)+strata(gender)+strata(centre)
                +eth +IDM+BMI
                +smoking+drinking4+Phy_act+fruit_g
                +HBP+chol_h+diabete_b+index
                +ASP+NASIDS+statin+ACEI+ARBs+beteb+vitamin+mineral+Health_R+illness_L
                ,data=data_IBS[data_IBS$Survtime_galst>2,])
HR <- round(exp(coef(fit)), 2)
CI <- round(exp(confint(fit)), 2)
P <- round(coef(summary(fit))[,5], 3)
colnames(CI) <- c("Lower", "Higher")
out1<- as.data.frame(cbind(HR, CI, P))
out1


#age
fit<- coxph(Surv(age1,Survtime_galst+age1,galst)~PPI_S0*age_fen
                +strata(gender)+strata(centre)
                +eth +IDM+bmic
                +smoking+drinking4+Phy_act+fruit_g
                +HBP+chol_h+diabete_b+index
                +ASP+NASIDS+statin+ACEI+ARBs+beteb+vitamin+mineral+Health_R+illness_L
                ,data=data_IBS[data_IBS$Survtime_galst>2,])
HR <- round(exp(coef(fit)), 2)
CI <- round(exp(confint(fit)), 2)
P <- round(coef(summary(fit))[,5], 3)
colnames(CI) <- c("Lower", "Higher")
out2<- as.data.frame(cbind(HR, CI, P))
out2


#activity

fit<- coxph(Surv(age1,Survtime_galst+age1,galst)~PPI_S0*act_fen
                +strata(agecat)+strata(gender)+strata(centre)
                +eth +IDM+BMI
                +smoking+drinking4+fruit_g
                +HBP+chol_h+diabete_b+index
```

```
                 +ASP+NASIDS+statin+ACEI+ARBs+beteb+vitamin+mineral+Health_R+illness_L
                 ,data=data_IBS[data_IBS$Survtime_galst>2,])
HR <- round(exp(coef(fit)), 2)
CI <- round(exp(confint(fit)), 2)
P <- round(coef(summary(fit))[,5], 3)
colnames(CI) <- c("Lower", "Higher")
out3<- as.data.frame(cbind(HR, CI, P))
out3


##smoking
fit<- coxph(Surv(age1,Survtime_galst+age1,galst)~PPI_S0*smk_fen
                 +strata(agecat)+strata(gender)+strata(centre)
                 +eth +IDM+bmic
                 +smoking+drinking4+Phy_act+fruit_g
                 +HBP+chol_h+diabete_b+index
                 +ASP+NASIDS+statin+ACEI+ARBs+beteb+vitamin+mineral+Health_R+illness_L
                 ,data=data_IBS[data_IBS$Survtime_galst>2,])
HR <- round(exp(coef(fit)), 2)
CI <- round(exp(confint(fit)), 2)
P <- round(coef(summary(fit))[,5], 3)
colnames(CI) <- c("Lower", "Higher")
out4<- as.data.frame(cbind(HR, CI, P))
out4


#drinking
fit<- coxph(Surv(age1,Survtime_galst+age1,galst)~PPI_S0*drk_fen
                 +strata(agecat)+strata(gender)+strata(centre)
                 +eth +IDM+bmic
                 +smoking+Phy_act+fruit_g
                 +HBP+chol_h+diabete_b+index
                 +ASP+NASIDS+statin+ACEI+ARBs+beteb+vitamin+mineral+Health_R+illness_L
                 ,data=data_IBS[data_IBS$Survtime_galst>2,])
HR <- round(exp(coef(fit)), 2)
CI <- round(exp(confint(fit)), 2)
P <- round(coef(summary(fit))[,5], 3)
colnames(CI) <- c("Lower", "Higher")
out5<- as.data.frame(cbind(HR, CI, P))
out5




#fruit and vegetable
fit<- coxph(Surv(age1,Survtime_galst+age1,galst)~PPI_S0*fruit_g
                 +strata(agecat)+strata(gender)+strata(centre)
                 +eth +IDM+bmic
                 +smoking+drinking4+Phy_act
```

```
                +HBP+chol_h+diabete_b+index
                +ASP+NASIDS+statin+ACEI+ARBs+beteb+vitamin+mineral+Health_R+illness_L
                ,data=data_IBS[data_IBS$Survtime_galst>2,])
HR <- round(exp(coef(fit)), 2)
CI <- round(exp(confint(fit)), 2)
P <- round(coef(summary(fit))[,5], 3)
colnames(CI) <- c("Lower", "Higher")
out6<- as.data.frame(cbind(HR, CI, P))
out6




#NASIDS
fit<- coxph(Surv(Survtime_galst,galst)~PPI_S0*NASIDS
                +strata(agecat)+strata(gender)+strata(centre)
                +eth +IDM+bmic
                +smoking+drinking4+Phy_act+fruit_g
                +HBP+chol_h+diabete_b+index
                +ASP+statin+ACEI+ARBs+beteb+vitamin+mineral+Health_R+illness_L
                ,data=data_IBS[data_IBS$Survtime_galst>2,])
HR <- round(exp(coef(fit)), 2)
CI <- round(exp(confint(fit)), 2)
P <- round(coef(summary(fit))[,5], 3)
colnames(CI) <- c("Lower", "Higher")
out7<- as.data.frame(cbind(HR, CI, P))
out7


#ASP
fit<- coxph(Surv(age1,Survtime_galst+age1,galst)~PPI_S0*ASP
                +strata(agecat)+strata(gender)+strata(centre)
                +eth +IDM+bmic
                +smoking+drinking4+Phy_act+fruit_g
                +HBP+chol_h+diabete_b+index
                +NASIDS+statin+ACEI+ARBs+beteb+vitamin+mineral+Health_R+illness_L
                ,data=data_IBS[data_IBS$Survtime_galst>2,])
HR <- round(exp(coef(fit)), 2)
CI <- round(exp(confint(fit)), 2)
P <- round(coef(summary(fit))[,5], 3)
colnames(CI) <- c("Lower", "Higher")
out8<- as.data.frame(cbind(HR, CI, P))
out8




#GERD
fit<- coxph(Surv(age1,Survtime_galst+age1,galst)~PPI_S0*gerd_b
```

```
                +strata(agecat)+strata(gender)+strata(centre)
                +eth +IDM+bmic
                +smoking+drinking4+Phy_act+fruit_g
                +HBP+chol_h+diabete_b
                +ASP+NASIDS+statin+ACEI+ARBs+beteb+vitamin+mineral+Health_R+illness_L
                ,data=data_IBS[data_IBS$Survtime_galst>2,])
HR <- round(exp(coef(fit)), 2)
CI <- round(exp(confint(fit)), 2)
P <- round(coef(summary(fit))[,5], 3)
colnames(CI) <- c("Lower", "Higher")
out9<- as.data.frame(cbind(HR, CI, P))
out9


p_interaction_ibd<- as.data.frame(rbind(out2,     out0,     out1,     out3,   out4,
                                                    out5,     out6,     out7,       out8,       out9))
p_interaction_ibd
write.table(p_interaction_ibd, file = "D:\\中山七院工作\\文献\\PPI\\cholecystitis\\p_interaction_ppi.csv", sep = ",",
                col.names = NA,qmethod = "double")




##### 22222222222222    H2RA 与 IBS main analyses -------------------------

#Modle1
fit<- coxph(Surv(age1,Survtime_galst+age1,galst)~h2ra_S0
                +strata(agecat)+strata(gender)+strata(centre)
                ,data=data_IBS[data_IBS$Survtime_galst>2,])
HR <- round(exp(coef(fit)), 2)
CI <- round(exp(confint(fit)), 2)
P <- round(coef(summary(fit))[,5], 3)
colnames(CI) <- c("Lower", "Higher")
out<- as.data.frame(cbind(HR, CI, P))
out1<- out[1,]
out1



#Modle2
fit<- coxph(Surv(age1,Survtime_galst+age1,galst)~h2ra_S0
                +strata(agecat)+strata(gender)+strata(centre)
                +eth +IDM+bmic
                +smoking+drinking4+Phy_act+fruit_g
                +HBP+chol_h+diabete_b+index
                +Health_R+illness_L
                ,data=data_IBS[data_IBS$Survtime_galst>2,])
HR <- round(exp(coef(fit)), 2)
```

```
CI <- round(exp(confint(fit)), 2)
P <- round(coef(summary(fit))[,5], 3)
colnames(CI) <- c("Lower", "Higher")
out<- as.data.frame(cbind(HR, CI, P))
out2<- out[1,]
out2


#Modle3
# centre    =data generated from different centre
fit<- coxph(Surv(age1,Survtime_galst+age1,galst)~h2ra_S0
            +strata(agecat)+strata(gender)+strata(centre)
            +eth +IDM+bmic
            +smoking+drinking4+Phy_act+fruit_g
            +HBP+chol_h+diabete_b+index
            +ASP+NASIDS+statin+ACEI+ARBs+beteb+vitamin+mineral+Health_R+illness_L
            ,data=data_IBS[data_IBS$Survtime_galst>2,])
HR <- round(exp(coef(fit)), 2)
CI <- round(exp(confint(fit)), 2)
P <- round(coef(summary(fit))[,5], 3)
colnames(CI) <- c("Lower", "Higher")
out<- as.data.frame(cbind(HR, CI, P))
out3<- out[1,]
out3


out_IBS<- as.data.frame(rbind(out1,out2,out3))
out_IBS
write.table(out_IBS, file = "D:\\中山七院工作\\文献\\PPI\\cholecystitis\\all_h2ra.csv", sep = ",",
            col.names = NA,qmethod = "double")


#for each type of H2RA and galst------------
ppi1<-table(data_IBS[data_IBS$Survtime_galst>2&data_IBS$non_H2RA=="No",]$galst)
ppi2<-aggregate(Survtime_galst~data_IBS[data_IBS$Survtime_galst>2,]$non_H2RA,
                data_IBS[data_IBS$Survtime_galst>2,],sum)
out0<- as.data.frame(cbind(ppi1,ppi2))
out0



rani01<-table(data_IBS[data_IBS$Survtime_galst>2,]$rani0,
              data_IBS[data_IBS$Survtime_galst>2,]$galst)
rani02<-aggregate(Survtime_galst~data_IBS[data_IBS$Survtime_galst>2,]$rani0,
                  data_IBS[data_IBS$Survtime_galst>2,],sum)
out1<- as.data.frame(cbind(rani01,rani02))
out1
```

```
H2_other1<-table(data_IBS[data_IBS$Survtime_galst>2,]$H2_other,
                              data_IBS[data_IBS$Survtime_galst>2,]$galst)
H2_other2<-aggregate(Survtime_galst~data_IBS[data_IBS$Survtime_galst>2,]$H2_other,
                                  data_IBS[data_IBS$Survtime_galst>2,],sum)
out2<- as.data.frame(cbind(H2_other1,H2_other2))
out2

h2_years_IBD<- as.data.frame(cbind(out0, out1, out2))
h2_years_IBD
write.table(h2_years_IBD, file = "D:\\中山七院工作\\文献\\PPI\\cholecystitis\\each_years_h2ra.csv", sep = ",",
                    col.names = NA,qmethod = "double")

##multivar-adjusted

# rani0 ="ranitidine at baseline"
fit<- coxph(Surv(age1,Survtime_galst+age1,galst)~rani0
                    +strata(agecat)+strata(gender)+strata(centre)
                    +eth +IDM+bmic
                    +smoking+drinking4+Phy_act+fruit_g
                    +HBP+chol_h+diabete_b+index
                    +ASP+NASIDS+statin+ACEI+ARBs+beteb+vitamin+mineral+Health_R+illness_L
                    ,data=data_IBS[data_IBS$Survtime_galst>2&(data_IBS$non_H2RA=="No"|data_IBS$rani0=="Yes"),])
HR <- round(exp(coef(fit)), 2)
CI <- round(exp(confint(fit)), 2)
P <- round(coef(summary(fit))[,5], 3)
colnames(CI) <- c("Lower", "Higher")
out<- as.data.frame(cbind(HR, CI, P))
out_rani<- out[1,]
out_rani

# H2_other="other H2RA at baseline"
fit<- coxph(Surv(age1,Survtime_galst+age1,galst)~H2_other
                    +strata(agecat)+strata(gender)+strata(centre)
                    +eth +IDM+bmic
                    +smoking+drinking4+Phy_act+fruit_g
                    +HBP+chol_h+diabete_b+index
                    +ASP+NASIDS+statin+ACEI+ARBs+beteb+vitamin+mineral+Health_R+illness_L
                    ,data=data_IBS[data_IBS$Survtime_galst>2&(data_IBS$non_H2RA=="No"|data_IBS$H2_other=="Yes"),])
HR <- round(exp(coef(fit)), 2)
CI <- round(exp(confint(fit)), 2)
P <- round(coef(summary(fit))[,5], 3)
colnames(CI) <- c("Lower", "Higher")
out<- as.data.frame(cbind(HR, CI, P))
out_other<- out[1,]
out_other


out_H2<- as.data.frame(rbind(out_rani,out_other))
out_H2
```

```
write.table(out_H2, file = "D:\\中山七院工作\\文献\\PPI\\cholecystitis\\each_H2RA_galst.csv", sep = ",",
            col.names = NA,qmethod = "double")




########subgroup analysis for galst --H2RA--------

data_IBS$bmi_fen<-1
data_IBS$bmi_fen[data_IBS$bmic!="obesity"]=0
data_IBS$bmi_fen<-factor(data_IBS$bmi_fen,levels = c(0,1),labels = c( "<30",">=30" ))


data_IBS$age_fen<-0
data_IBS$age_fen[data_IBS$age1 >=60]=1
data_IBS$age_fen<-factor(data_IBS$age_fen,levels = c(0,1),labels = c( "<60",">=60" ))


data_IBS$smk_fen<-0
data_IBS$smk_fen[data_IBS$smoking!="Never"]=1
data_IBS$smk_fen<-factor(data_IBS$smk_fen,levels = c(0,1),labels = c( "No smoker","Smoker" ))

data_IBS$drk_fen<-1
data_IBS$drk_fen[data_IBS$drinking=="Never"|data_IBS$drinking=="unkonwn/missing"]=0
data_IBS$drk_fen<-factor(data_IBS$drk_fen,levels = c(0,1),labels = c( "No drinker","Drinker" ))


data_IBS$act_fen<-0
data_IBS$act_fen[data_IBS$MET >=1771]=1
data_IBS$act_fen<-factor(data_IBS$act_fen,levels = c(0,1),labels = c( "activity < median","activity >= median" ))



#####For Male
fit<- coxph(Surv(age1,Survtime_galst+age1,galst)~h2ra_S0
            +strata(agecat)+strata(centre)
            +eth +IDM+bmic
            +smoking+drinking4+Phy_act+fruit_g
            +HBP+chol_h+diabete_b+index
            +ASP+NASIDS+statin+ACEI+ARBs+beteb+vitamin+mineral+Health_R+illness_L
            ,data=data_IBS[data_IBS$Survtime_galst>2&data_IBS$gender=="Male",])
HR <- round(exp(coef(fit)), 2)
CI <- round(exp(confint(fit)), 2)
P <- round(coef(summary(fit))[,5], 3)
colnames(CI) <- c("Lower", "Higher")
out<- as.data.frame(cbind(HR, CI, P))
out_male<- out[1,]
out_male
```

```
#####For Female
fit<- coxph(Surv(age1,Survtime_galst+age1,galst)~h2ra_S0
            +strata(agecat)+strata(centre)
            +eth +IDM+bmic
            +smoking+drinking4+Phy_act+fruit_g
            +HBP+chol_h+diabete_b+index
            +ASP+NASIDS+statin+ACEI+ARBs+beteb+vitamin+mineral+Health_R+illness_L
            +menopause1+HRT+OCT
            ,data=data_IBS[data_IBS$Survtime_galst>2&data_IBS$gender=="Female",])
HR <- round(exp(coef(fit)), 2)
CI <- round(exp(confint(fit)), 2)
P <- round(coef(summary(fit))[,5], 3)
colnames(CI) <- c("Lower", "Higher")
out<- as.data.frame(cbind(HR, CI, P))
out_female<- out[1,]
out_female


#bmi_fen
fit<- coxph(Surv(age1,Survtime_galst+age1,galst)~h2ra_S0
            +strata(agecat)+strata(gender)+strata(centre)
            +eth +IDM+BMI
            +smoking+drinking4+Phy_act+fruit_g
            +HBP+chol_h+diabete_b+index
            +ASP+NASIDS+statin+ACEI+ARBs+beteb+vitamin+mineral+Health_R+illness_L
            ,data=data_IBS[data_IBS$Survtime_galst>2&data_IBS$bmi_fen=="<30",])
HR <- round(exp(coef(fit)), 2)
CI <- round(exp(confint(fit)), 2)
P <- round(coef(summary(fit))[,5], 3)
colnames(CI) <- c("Lower", "Higher")
out<- as.data.frame(cbind(HR, CI, P))
bmi_out0<- out[1,]
bmi_out0


fit<- coxph(Surv(age1,Survtime_galst+age1,galst)~h2ra_S0
            +strata(agecat)+strata(gender)+strata(centre)
            +eth +IDM+BMI
            +smoking+drinking4+Phy_act+fruit_g
            +HBP+chol_h+diabete_b+index
            +ASP+NASIDS+statin+ACEI+ARBs+beteb+vitamin+mineral+Health_R+illness_L
            ,data=data_IBS[data_IBS$Survtime_galst>2&data_IBS$bmi_fen==">=30",])
HR <- round(exp(coef(fit)), 2)
CI <- round(exp(confint(fit)), 2)
P <- round(coef(summary(fit))[,5], 3)
colnames(CI) <- c("Lower", "Higher")
out<- as.data.frame(cbind(HR, CI, P))
bmi_out1<- out[1,]
bmi_out1
```

```
#age
fit<- coxph(Surv(age1,Survtime_galst+age1,galst)~h2ra_S0
            +strata(agecat)+strata(gender)+strata(centre)
            +eth +IDM+bmic
            +smoking+drinking4+Phy_act+fruit_g
            +HBP+chol_h+diabete_b+index
            +ASP+NASIDS+statin+ACEI+ARBs+beteb+vitamin+mineral+Health_R+illness_L
            ,data=data_IBS[data_IBS$Survtime_galst>2&data_IBS$age_fen=="<60",])
HR <- round(exp(coef(fit)), 2)
CI <- round(exp(confint(fit)), 2)
P <- round(coef(summary(fit))[,5], 3)
colnames(CI) <- c("Lower", "Higher")
out<- as.data.frame(cbind(HR, CI, P))
age_out0<- out[1,]
age_out0


fit<- coxph(Surv(age1,Survtime_galst+age1,galst)~h2ra_S0
            +strata(agecat)+strata(gender)+strata(centre)
            +eth +IDM+bmic
            +smoking+drinking4+Phy_act+fruit_g
            +HBP+chol_h+diabete_b+index
            +ASP+NASIDS+statin+ACEI+ARBs+beteb+vitamin+mineral+Health_R+illness_L
            ,data=data_IBS[data_IBS$Survtime_galst>2&data_IBS$age_fen==">=60",])
HR <- round(exp(coef(fit)), 2)
CI <- round(exp(confint(fit)), 2)
P <- round(coef(summary(fit))[,5], 3)
colnames(CI) <- c("Lower", "Higher")
out<- as.data.frame(cbind(HR, CI, P))
age_out1<- out[1,]
age_out1




#activity

fit<- coxph(Surv(age1,Survtime_galst+age1,galst)~h2ra_S0
            +strata(agecat)+strata(gender)+strata(centre)
            +eth +IDM+bmic
            +smoking+drinking4+fruit_g
            +HBP+chol_h+diabete_b+index
            +ASP+NASIDS+statin+ACEI+ARBs+beteb+vitamin+mineral+Health_R+illness_L
            ,data=data_IBS[data_IBS$Survtime_galst>2&data_IBS$act_fen=="activity < median",])
HR <- round(exp(coef(fit)), 2)
CI <- round(exp(confint(fit)), 2)
P <- round(coef(summary(fit))[,5], 3)
```

```
colnames(CI) <- c("Lower", "Higher")
out<- as.data.frame(cbind(HR, CI, P))
act_out0<- out[1,]
act_out0


fit<- coxph(Surv(age1,Survtime_galst+age1,galst)~h2ra_S0
            +strata(agecat)+strata(gender)+strata(centre)
            +eth +IDM+bmic
            +smoking+drinking4+fruit_g
            +HBP+chol_h+diabete_b+index
            +ASP+NASIDS+statin+ACEI+ARBs+beteb+vitamin+mineral+Health_R+illness_L
            ,data=data_IBS[data_IBS$Survtime_galst>2&data_IBS$act_fen=="activity >= median",])
HR <- round(exp(coef(fit)), 2)
CI <- round(exp(confint(fit)), 2)
P <- round(coef(summary(fit))[,5], 3)
colnames(CI) <- c("Lower", "Higher")
out<- as.data.frame(cbind(HR, CI, P))
act_out1<- out[1,]
act_out1


##smoking
fit<- coxph(Surv(age1,Survtime_galst+age1,galst)~h2ra_S0
            +strata(agecat)+strata(gender)+strata(centre)
            +eth +IDM+bmic
            +drinking4+Phy_act+fruit_g
            +HBP+chol_h+diabete_b+index
            +ASP+NASIDS+statin+ACEI+ARBs+beteb+vitamin+mineral+Health_R+illness_L
            ,data=data_IBS[data_IBS$Survtime_galst>2&data_IBS$smk_fen=="No smoker",])
HR <- round(exp(coef(fit)), 2)
CI <- round(exp(confint(fit)), 2)
P <- round(coef(summary(fit))[,5], 3)
colnames(CI) <- c("Lower", "Higher")
out<- as.data.frame(cbind(HR, CI, P))
smk_out0<- out[1,]
smk_out0


fit<- coxph(Surv(age1,Survtime_galst+age1,galst)~h2ra_S0
            +strata(agecat)+strata(gender)+strata(centre)
            +eth +IDM+bmic
            +drinking4+Phy_act+fruit_g
            +HBP+chol_h+diabete_b+index
            +ASP+NASIDS+statin+ACEI+ARBs+beteb+vitamin+mineral+Health_R+illness_L
            ,data=data_IBS[data_IBS$Survtime_galst>2&data_IBS$smk_fen=="Smoker",])
HR <- round(exp(coef(fit)), 2)
CI <- round(exp(confint(fit)), 2)
P <- round(coef(summary(fit))[,5], 3)
```

```
colnames(CI) <- c("Lower", "Higher")
out<- as.data.frame(cbind(HR, CI, P))
smk_out1<- out[1,]
smk_out1



#drinking
fit<- coxph(Surv(age1,Survtime_galst+age1,galst)~h2ra_S0
            +strata(agecat)+strata(gender)+strata(centre)
            +eth +IDM+bmic
            +smoking+Phy_act+fruit_g
            +HBP+chol_h+diabete_b+index
            +ASP+NASIDS+statin+ACEI+ARBs+beteb+vitamin+mineral+Health_R+illness_L
            ,data=data_IBS[data_IBS$Survtime_galst>2&data_IBS$drk_fen=="No drinker",])
HR <- round(exp(coef(fit)), 2)
CI <- round(exp(confint(fit)), 2)
P <- round(coef(summary(fit))[,5], 3)
colnames(CI) <- c("Lower", "Higher")
out<- as.data.frame(cbind(HR, CI, P))
drk_out0<- out[1,]
drk_out0


fit<- coxph(Surv(age1,Survtime_galst+age1,galst)~h2ra_S0
            +strata(agecat)+strata(gender)+strata(centre)
            +eth +IDM+bmic
            +smoking+Phy_act+fruit_g
            +HBP+chol_h+diabete_b+index
            +ASP+NASIDS+statin+ACEI+ARBs+beteb+vitamin+mineral+Health_R+illness_L
            ,data=data_IBS[data_IBS$Survtime_galst>2&data_IBS$drk_fen=="Drinker",])
HR <- round(exp(coef(fit)), 2)
CI <- round(exp(confint(fit)), 2)
P <- round(coef(summary(fit))[,5], 3)
colnames(CI) <- c("Lower", "Higher")
out<- as.data.frame(cbind(HR, CI, P))
drk_out1<- out[1,]
drk_out1




#fruit and vegetable
fit<- coxph(Surv(age1,Survtime_galst+age1,galst)~h2ra_S0
            +strata(agecat)+strata(gender)+strata(centre)
            +eth +IDM+bmic
            +smoking+drinking4+Phy_act
            +HBP+chol_h+diabete_b+index
            +ASP+NASIDS+statin+ACEI+ARBs+beteb+vitamin+mineral+Health_R+illness_L
```

```
                    ,data=data_IBS[data_IBS$Survtime_galst>2&data_IBS$fruit_g=="No",])
HR <- round(exp(coef(fit)), 2)
CI <- round(exp(confint(fit)), 2)
P <- round(coef(summary(fit))[,5], 3)
colnames(CI) <- c("Lower", "Higher")
out<- as.data.frame(cbind(HR, CI, P))
out_fru0<- out[1,]
out_fru0


fit<- coxph(Surv(age1,Survtime_galst+age1,galst)~h2ra_S0
                    +strata(agecat)+strata(gender)+strata(centre)
                    +eth +IDM+bmic
                    +smoking+drinking4+Phy_act
                    +HBP+chol_h+diabete_b+index
                    +ASP+NASIDS+statin+ACEI+ARBs+beteb+vitamin+mineral+Health_R+illness_L
                    ,data=data_IBS[data_IBS$Survtime_galst>2&data_IBS$fruit_g=="Yes",])
HR <- round(exp(coef(fit)), 2)
CI <- round(exp(confint(fit)), 2)
P <- round(coef(summary(fit))[,5], 3)
colnames(CI) <- c("Lower", "Higher")
out<- as.data.frame(cbind(HR, CI, P))
out_fru1<- out[1,]
out_fru1



#NASIDS
fit<- coxph(Surv(age1,Survtime_galst+age1,galst)~h2ra_S0
                    +strata(agecat)+strata(gender)+strata(centre)
                    +eth +IDM+bmic
                    +smoking+drinking4+Phy_act+fruit_g
                    +HBP+chol_h+diabete_b+index
                    +ASP+statin+ACEI+ARBs+beteb+vitamin+mineral+Health_R+illness_L
                    ,data=data_IBS[data_IBS$Survtime_galst>2&data_IBS$NASIDS==0,])
HR <- round(exp(coef(fit)), 2)
CI <- round(exp(confint(fit)), 2)
P <- round(coef(summary(fit))[,5], 3)
colnames(CI) <- c("Lower", "Higher")
out<- as.data.frame(cbind(HR, CI, P))
NASIDS_out0<- out[1,]
NASIDS_out0


fit<- coxph(Surv(age1,Survtime_galst+age1,galst)~h2ra_S0
                    +strata(agecat)+strata(gender)+strata(centre)
                    +eth +IDM+bmic
                    +smoking+drinking4+Phy_act+fruit_g
                    +HBP+chol_h+diabete_b+index
```

```
                      +ASP+statin+ACEI+ARBs+beteb+vitamin+mineral+Health_R+illness_L
                      ,data=data_IBS[data_IBS$Survtime_galst>2&data_IBS$NASIDS==1,])
HR <- round(exp(coef(fit)), 2)
CI <- round(exp(confint(fit)), 2)
P <- round(coef(summary(fit))[,5], 3)
colnames(CI) <- c("Lower", "Higher")
out<- as.data.frame(cbind(HR, CI, P))
NASIDS_out1<- out[1,]
NASIDS_out1


#ASP
fit<- coxph(Surv(age1,Survtime_galst+age1,galst)~h2ra_S0
                      +strata(agecat)+strata(gender)+strata(centre)
                      +eth +IDM+bmic
                      +smoking+drinking4+Phy_act+fruit_g
                      +HBP+chol_h+diabete_b+index
                      +NASIDS+statin+ACEI+ARBs+beteb+vitamin+mineral+Health_R+illness_L
                      ,data=data_IBS[data_IBS$Survtime_galst>2&data_IBS$ASP==0,])
HR <- round(exp(coef(fit)), 2)
CI <- round(exp(confint(fit)), 2)
P <- round(coef(summary(fit))[,5], 3)
colnames(CI) <- c("Lower", "Higher")
out<- as.data.frame(cbind(HR, CI, P))
ASP_out0<- out[1,]
ASP_out0


fit<- coxph(Surv(age1,Survtime_galst+age1,galst)~h2ra_S0
                      +strata(agecat)+strata(gender)+strata(centre)
                      +eth +IDM+bmic
                      +smoking+drinking4+Phy_act+fruit_g
                      +HBP+chol_h+diabete_b+index
                      +NASIDS+statin+ACEI+ARBs+beteb+vitamin+mineral+Health_R+illness_L
                      ,data=data_IBS[data_IBS$Survtime_galst>2&data_IBS$ASP==1,])
HR <- round(exp(coef(fit)), 2)
CI <- round(exp(confint(fit)), 2)
P <- round(coef(summary(fit))[,5], 3)
colnames(CI) <- c("Lower", "Higher")
out<- as.data.frame(cbind(HR, CI, P))
ASP_out1<- out[1,]
ASP_out1




#GERD
fit<- coxph(Surv(age1,Survtime_galst+age1,galst)~h2ra_S0
                      +strata(agecat)+strata(gender)+strata(centre)
```

```
                    +eth +IDM+bmic
                    +smoking+drinking4+Phy_act+fruit_g
                    +HBP+chol_h+diabete_b
                    +ASP+NASIDS+statin+ACEI+ARBs+beteb+vitamin+mineral+Health_R+illness_L
                    ,data=data_IBS[data_IBS$Survtime_galst>2&data_IBS$gerd_b==0,])
HR <- round(exp(coef(fit)), 2)
CI <- round(exp(confint(fit)), 2)
P <- round(coef(summary(fit))[,5], 3)
colnames(CI) <- c("Lower", "Higher")
out<- as.data.frame(cbind(HR, CI, P))
gerd_out0<- out[1,]
gerd_out0


fit<- coxph(Surv(age1,Survtime_galst+age1,galst)~h2ra_S0
                    +strata(agecat)+strata(gender)+strata(centre)
                    +eth +IDM+bmic
                    +smoking+drinking4+Phy_act+fruit_g
                    +HBP+chol_h+diabete_b
                    +ASP+NASIDS+statin+ACEI+ARBs+beteb+vitamin+mineral+Health_R+illness_L
                    ,data=data_IBS[data_IBS$Survtime_galst>2&data_IBS$gerd_b==1,])
HR <- round(exp(coef(fit)), 2)
CI <- round(exp(confint(fit)), 2)
P <- round(coef(summary(fit))[,5], 3)
colnames(CI) <- c("Lower", "Higher")
out<- as.data.frame(cbind(HR, CI, P))
gerd_out1<- out[1,]
gerd_out1

subgroup_IBD<- as.data.frame(rbind(out_male,out_female,age_out0,age_out1,bmi_out0,bmi_out1,
                                   smk_out0,smk_out1,drk_out0,drk_out1,
                                   act_out0,act_out1,out_fru0,out_fru1,
                                   NASIDS_out0,NASIDS_out1,ASP_out0,ASP_out1,
                                   gerd_out0,gerd_out1))
subgroup_IBD

write.table(subgroup_IBD, file = "D:\\中山七院工作\\文献\\PPI\\cholecystitis\\subgroup_h2ra.csv", sep = ",",
                    col.names = NA,qmethod = "double")



### p interaction for H2RA--galst association--------

#gender
fit<- coxph(Surv(age1,Survtime_galst+age1,galst)~h2ra_S0*gender
                    +strata(agecat)+strata(centre)
                    +eth +IDM+bmic
                    +smoking+drinking4+Phy_act+fruit_g
                    +HBP+chol_h+diabete_b+index
```

```
                +ASP+NASIDS+statin+ACEI+ARBs+beteb+vitamin+mineral+Health_R+illness_L
                ,data=data_IBS[data_IBS$Survtime_galst>2,])
HR <- round(exp(coef(fit)), 2)
CI <- round(exp(confint(fit)), 2)
P <- round(coef(summary(fit))[,5], 3)
colnames(CI) <- c("Lower", "Higher")
out0<- as.data.frame(cbind(HR, CI, P))
out0



#bmi_fen
fit<- coxph(Surv(age1,Survtime_galst+age1,galst)~h2ra_S0*bmi_fen
                +strata(agecat)+strata(gender)+strata(centre)
                +eth +IDM+BMI
                +smoking+drinking4+Phy_act+fruit_g
                +HBP+chol_h+diabete_b+index
                +ASP+NASIDS+statin+ACEI+ARBs+beteb+vitamin+mineral+Health_R+illness_L
                ,data=data_IBS[data_IBS$Survtime_galst>2,])
HR <- round(exp(coef(fit)), 2)
CI <- round(exp(confint(fit)), 2)
P <- round(coef(summary(fit))[,5], 3)
colnames(CI) <- c("Lower", "Higher")
out1<- as.data.frame(cbind(HR, CI, P))
out1


#age
fit<- coxph(Surv(age1,Survtime_galst+age1,galst)~h2ra_S0*age_fen
                +strata(gender)+strata(centre)
                +eth +IDM+bmic
                +smoking+drinking4+Phy_act+fruit_g
                +HBP+chol_h+diabete_b+index
                +ASP+NASIDS+statin+ACEI+ARBs+beteb+vitamin+mineral+Health_R+illness_L
                ,data=data_IBS[data_IBS$Survtime_galst>2,])
HR <- round(exp(coef(fit)), 2)
CI <- round(exp(confint(fit)), 2)
P <- round(coef(summary(fit))[,5], 3)
colnames(CI) <- c("Lower", "Higher")
out2<- as.data.frame(cbind(HR, CI, P))
out2


#activity

fit<- coxph(Surv(age1,Survtime_galst+age1,galst)~h2ra_S0*act_fen
                +strata(agecat)+strata(gender)+strata(centre)
                +eth +IDM+BMI
                +smoking+drinking4+fruit_g
```

```
             +HBP+chol_h+diabete_b+index
             +ASP+NASIDS+statin+ACEI+ARBs+beteb+vitamin+mineral+Health_R+illness_L
             ,data=data_IBS[data_IBS$Survtime_galst>2,])
HR <- round(exp(coef(fit)), 2)
CI <- round(exp(confint(fit)), 2)
P <- round(coef(summary(fit))[,5], 3)
colnames(CI) <- c("Lower", "Higher")
out3<- as.data.frame(cbind(HR, CI, P))
out3


##smoking
fit<- coxph(Surv(age1,Survtime_galst+age1,galst)~h2ra_S0*smk_fen
             +strata(agecat)+strata(gender)+strata(centre)
             +eth +IDM+bmic
             +smoking+drinking4+Phy_act+fruit_g
             +HBP+chol_h+diabete_b+index
             +ASP+NASIDS+statin+ACEI+ARBs+beteb+vitamin+mineral+Health_R+illness_L
             ,data=data_IBS[data_IBS$Survtime_galst>2,])
HR <- round(exp(coef(fit)), 2)
CI <- round(exp(confint(fit)), 2)
P <- round(coef(summary(fit))[,5], 3)
colnames(CI) <- c("Lower", "Higher")
out4<- as.data.frame(cbind(HR, CI, P))
out4


#drinking
fit<- coxph(Surv(age1,Survtime_galst+age1,galst)~h2ra_S0*drk_fen
             +strata(agecat)+strata(gender)+strata(centre)
             +eth +IDM+bmic
             +smoking+Phy_act+fruit_g
             +HBP+chol_h+diabete_b+index
             +ASP+NASIDS+statin+ACEI+ARBs+beteb+vitamin+mineral+Health_R+illness_L
             ,data=data_IBS[data_IBS$Survtime_galst>2,])
HR <- round(exp(coef(fit)), 2)
CI <- round(exp(confint(fit)), 2)
P <- round(coef(summary(fit))[,5], 3)
colnames(CI) <- c("Lower", "Higher")
out5<- as.data.frame(cbind(HR, CI, P))
out5




#fruit and vegetable
fit<- coxph(Surv(age1,Survtime_galst+age1,galst)~h2ra_S0*fruit_g
             +strata(agecat)+strata(gender)+strata(centre)
             +eth +IDM+bmic
```

```
                  +smoking+drinking4+Phy_act
                  +HBP+chol_h+diabete_b+index
                  +ASP+NASIDS+statin+ACEI+ARBs+beteb+vitamin+mineral+Health_R+illness_L
                  ,data=data_IBS[data_IBS$Survtime_galst>2,])
HR <- round(exp(coef(fit)), 2)
CI <- round(exp(confint(fit)), 2)
P <- round(coef(summary(fit))[,5], 3)
colnames(CI) <- c("Lower", "Higher")
out6<- as.data.frame(cbind(HR, CI, P))
out6




#NASIDS
fit<- coxph(Surv(Survtime_galst,galst)~h2ra_S0*NASIDS
                  +strata(agecat)+strata(gender)+strata(centre)
                  +eth +IDM+bmic
                  +smoking+drinking4+Phy_act+fruit_g
                  +HBP+chol_h+diabete_b+index
                  +ASP+statin+ACEI+ARBs+beteb+vitamin+mineral+Health_R+illness_L
                  ,data=data_IBS[data_IBS$Survtime_galst>2,])
HR <- round(exp(coef(fit)), 2)
CI <- round(exp(confint(fit)), 2)
P <- round(coef(summary(fit))[,5], 3)
colnames(CI) <- c("Lower", "Higher")
out7<- as.data.frame(cbind(HR, CI, P))
out7


#ASP
fit<- coxph(Surv(age1,Survtime_galst+age1,galst)~h2ra_S0*ASP
                  +strata(agecat)+strata(gender)+strata(centre)
                  +eth +IDM+bmic
                  +smoking+drinking4+Phy_act+fruit_g
                  +HBP+chol_h+diabete_b+index
                  +NASIDS+statin+ACEI+ARBs+beteb+vitamin+mineral+Health_R+illness_L
                  ,data=data_IBS[data_IBS$Survtime_galst>2,])
HR <- round(exp(coef(fit)), 2)
CI <- round(exp(confint(fit)), 2)
P <- round(coef(summary(fit))[,5], 3)
colnames(CI) <- c("Lower", "Higher")
out8<- as.data.frame(cbind(HR, CI, P))
out8




#GERD
```

```
fit<- coxph(Surv(age1,Survtime_galst+age1,galst)~h2ra_S0*gerd_b
            +strata(agecat)+strata(gender)+strata(centre)
            +eth +IDM+bmic
            +smoking+drinking4+Phy_act+fruit_g
            +HBP+chol_h+diabete_b
            +ASP+NASIDS+statin+ACEI+ARBs+beteb+vitamin+mineral+Health_R+illness_L
            ,data=data_IBS[data_IBS$Survtime_galst>2,])
HR <- round(exp(coef(fit)), 2)
CI <- round(exp(confint(fit)), 2)
P <- round(coef(summary(fit))[,5], 3)
colnames(CI) <- c("Lower", "Higher")
out9<- as.data.frame(cbind(HR, CI, P))
out9


p_interaction_ibd<- as.data.frame(rbind(out2,    out0,    out1,    out3,   out4,
                                         out5,    out6,    out7,    out8,    out9))
p_interaction_ibd
write.table(p_interaction_ibd, file = "D:\\中山七院工作\\文献\\PPI\\cholecystitis\\p_interaction_h2ra.csv", sep = ",",
            col.names = NA,qmethod = "double")





#######calculate the cases and Person-years for subgroup-----

#Subgroups for galst---ppi
gender1<-table(data_IBS[data_IBS$Survtime_galst>2&data_IBS$PPI_S0=="Yes",]$gender,data_IBS[data_IBS$Survtime_galst>2&data_IBS$PPI_S0=="Yes",]$galst)
gender2<-aggregate(Survtime_galst~data_IBS[data_IBS$Survtime_galst>2&data_IBS$PPI_S0=="Yes",]$gender,data_IBS[data_IBS$Survtime_galst>2&data_IBS$PPI_S0=="Yes",],sum)
out1<- as.data.frame(cbind(gender1,gender2))
out1

age1<-table(data_IBS[data_IBS$Survtime_galst>2&data_IBS$PPI_S0=="Yes",]$age_fen,data_IBS[data_IBS$Survtime_galst>2&data_IBS$PPI_S0=="Yes",]$galst)
age2<-aggregate(Survtime_galst~data_IBS[data_IBS$Survtime_galst>2&data_IBS$PPI_S0=="Yes",]$age_fen,data_IBS[data_IBS$Survtime_galst>2&data_IBS$PPI_S0=="Yes",],sum)
out2<- as.data.frame(cbind(age1,age2))
out2


bmi1<-table(data_IBS[data_IBS$Survtime_galst>2&data_IBS$PPI_S0=="Yes",]$bmi_fen,data_IBS[data_IBS$Survtime_galst>2&data_IBS$PPI_S0=="Yes",]$galst)
bmi2<-aggregate(Survtime_galst~data_IBS[data_IBS$Survtime_galst>2&data_IBS$PPI_S0=="Yes",]$bmi_fen,data_IBS[data_IBS$Survtime_galst>2&data_IBS$PPI_S0=="Yes",],sum)
out3<- as.data.frame(cbind(bmi1,bmi2))
out3
```

```
smk1<-table(data_IBS[data_IBS$Survtime_galst>2&data_IBS$PPI_S0=="Yes",]$smk_fen,data_IBS[data_IBS$Survtime_galst>2&data_IBS$PPI_S0
=="Yes",]$galst)
smk2<-aggregate(Survtime_galst~data_IBS[data_IBS$Survtime_galst>2&data_IBS$PPI_S0=="Yes",]$smk_fen,data_IBS[data_IBS$Survtime_galst
>2&data_IBS$PPI_S0=="Yes",],sum)
out4<- as.data.frame(cbind(smk1,smk2))
out4

drk1<-table(data_IBS[data_IBS$Survtime_galst>2&data_IBS$PPI_S0=="Yes",]$drk_fen,data_IBS[data_IBS$Survtime_galst>2&data_IBS$PPI_S0=
="Yes",]$galst)
drk2<-aggregate(Survtime_galst~data_IBS[data_IBS$Survtime_galst>2&data_IBS$PPI_S0=="Yes",]$drk_fen,data_IBS[data_IBS$Survtime_galst>
2&data_IBS$PPI_S0=="Yes",],sum)
out5<- as.data.frame(cbind(drk1,drk2))
out5


act1<-table(data_IBS[data_IBS$Survtime_galst>2&data_IBS$PPI_S0=="Yes",]$act_fen,data_IBS[data_IBS$Survtime_galst>2&data_IBS$PPI_S0=
="Yes",]$galst)
act2<-aggregate(Survtime_galst~data_IBS[data_IBS$Survtime_galst>2&data_IBS$PPI_S0=="Yes",]$act_fen,data_IBS[data_IBS$Survtime_galst>
2&data_IBS$PPI_S0=="Yes",],sum)
out6<- as.data.frame(cbind(act1,act2))
out6


fru1<-table(data_IBS[data_IBS$Survtime_galst>2&data_IBS$PPI_S0=="Yes",]$fruit_g,data_IBS[data_IBS$Survtime_galst>2&data_IBS$PPI_S0==
"Yes",]$galst)
fru2<-aggregate(Survtime_galst~data_IBS[data_IBS$Survtime_galst>2&data_IBS$PPI_S0=="Yes",]$fruit_g,data_IBS[data_IBS$Survtime_galst>2
&data_IBS$PPI_S0=="Yes",],sum)
out7<- as.data.frame(cbind(fru1,fru2))
out7


nas1<-table(data_IBS[data_IBS$Survtime_galst>2&data_IBS$PPI_S0=="Yes",]$NASIDS,data_IBS[data_IBS$Survtime_galst>2&data_IBS$PPI_S0=
="Yes",]$galst)
nas2<-aggregate(Survtime_galst~data_IBS[data_IBS$Survtime_galst>2&data_IBS$PPI_S0=="Yes",]$NASIDS,data_IBS[data_IBS$Survtime_galst>
2&data_IBS$PPI_S0=="Yes",],sum)
out8<- as.data.frame(cbind(nas1,nas2))
out8


asp1<-table(data_IBS[data_IBS$Survtime_galst>2&data_IBS$PPI_S0=="Yes",]$ASP,data_IBS[data_IBS$Survtime_galst>2&data_IBS$PPI_S0=="Ye
s",]$galst)
asp2<-aggregate(Survtime_galst~data_IBS[data_IBS$Survtime_galst>2&data_IBS$PPI_S0=="Yes",]$ASP,data_IBS[data_IBS$Survtime_galst>2&d
ata_IBS$PPI_S0=="Yes",],sum)
out9<- as.data.frame(cbind(asp1,asp2))
out9


gerd1<-table(data_IBS[data_IBS$Survtime_galst>2&data_IBS$PPI_S0=="Yes",]$gerd_b,data_IBS[data_IBS$Survtime_galst>2&data_IBS$PPI_S0=
```

```
="Yes",]$galst)
gerd2<-aggregate(Survtime_galst~data_IBS[data_IBS$Survtime_galst>2&data_IBS$PPI_S0=="Yes",]$gerd_b,data_IBS[data_IBS$Survtime_galst>
2&data_IBS$PPI_S0=="Yes",],sum)
out10<- as.data.frame(cbind(gerd1,gerd2))
out10


pearson_years_ibd<- as.data.frame(cbind(out1, out2,      out3,         out4,         out5,
                                                            out6,     out7, out8,         out9,         out10))
pearson_years_ibd

write.table(pearson_years_ibd, file = "D:\\中山七院工作\\文献\\PPI\\cholecystitis\\years_subgroup_ppi.csv", sep = ",",
                  col.names = NA,qmethod = "double")


#Subgroups for galst——for h2ra
gender1<-table(data_IBS[data_IBS$Survtime_galst>2&data_IBS$h2ra_S0=="Yes",]$gender,data_IBS[data_IBS$Survtime_galst>2&data_IBS$h2r
a_S0=="Yes",]$galst)
gender2<-aggregate(Survtime_galst~data_IBS[data_IBS$Survtime_galst>2&data_IBS$h2ra_S0=="Yes",]$gender,data_IBS[data_IBS$Survtime_g
alst>2&data_IBS$h2ra_S0=="Yes",],sum)
out1<- as.data.frame(cbind(gender1,gender2))
out1

age1<-table(data_IBS[data_IBS$Survtime_galst>2&data_IBS$h2ra_S0=="Yes",]$age_fen,data_IBS[data_IBS$Survtime_galst>2&data_IBS$h2ra_
S0=="Yes",]$galst)
age2<-aggregate(Survtime_galst~data_IBS[data_IBS$Survtime_galst>2&data_IBS$h2ra_S0=="Yes",]$age_fen,data_IBS[data_IBS$Survtime_gals
t>2&data_IBS$h2ra_S0=="Yes",],sum)
out2<- as.data.frame(cbind(age1,age2))
out2


bmi1<-table(data_IBS[data_IBS$Survtime_galst>2&data_IBS$h2ra_S0=="Yes",]$bmi_fen,data_IBS[data_IBS$Survtime_galst>2&data_IBS$h2ra_
S0=="Yes",]$galst)
bmi2<-aggregate(Survtime_galst~data_IBS[data_IBS$Survtime_galst>2&data_IBS$h2ra_S0=="Yes",]$bmi_fen,data_IBS[data_IBS$Survtime_gals
t>2&data_IBS$h2ra_S0=="Yes",],sum)
out3<- as.data.frame(cbind(bmi1,bmi2))
out3

smk1<-table(data_IBS[data_IBS$Survtime_galst>2&data_IBS$h2ra_S0=="Yes",]$smk_fen,data_IBS[data_IBS$Survtime_galst>2&data_IBS$h2ra
_S0=="Yes",]$galst)
smk2<-aggregate(Survtime_galst~data_IBS[data_IBS$Survtime_galst>2&data_IBS$h2ra_S0=="Yes",]$smk_fen,data_IBS[data_IBS$Survtime_gal
st>2&data_IBS$h2ra_S0=="Yes",],sum)
out4<- as.data.frame(cbind(smk1,smk2))
out4

drk1<-table(data_IBS[data_IBS$Survtime_galst>2&data_IBS$h2ra_S0=="Yes",]$drk_fen,data_IBS[data_IBS$Survtime_galst>2&data_IBS$h2ra_S
0=="Yes",]$galst)
drk2<-aggregate(Survtime_galst~data_IBS[data_IBS$Survtime_galst>2&data_IBS$h2ra_S0=="Yes",]$drk_fen,data_IBS[data_IBS$Survtime_galst
>2&data_IBS$h2ra_S0=="Yes",],sum)
```

```
out5<- as.data.frame(cbind(drk1,drk2))
out5


act1<-table(data_IBS[data_IBS$Survtime_galst>2&data_IBS$h2ra_S0=="Yes",]$act_fen,data_IBS[data_IBS$Survtime_galst>2&data_IBS$h2ra_S
0=="Yes",]$galst)
act2<-aggregate(Survtime_galst~data_IBS[data_IBS$Survtime_galst>2&data_IBS$h2ra_S0=="Yes",]$act_fen,data_IBS[data_IBS$Survtime_galst
>2&data_IBS$h2ra_S0=="Yes",],sum)
out6<- as.data.frame(cbind(act1,act2))
out6


fru1<-table(data_IBS[data_IBS$Survtime_galst>2&data_IBS$h2ra_S0=="Yes",]$fruit_g,data_IBS[data_IBS$Survtime_galst>2&data_IBS$h2ra_S0
=="Yes",]$galst)
fru2<-aggregate(Survtime_galst~data_IBS[data_IBS$Survtime_galst>2&data_IBS$h2ra_S0=="Yes",]$fruit_g,data_IBS[data_IBS$Survtime_galst>
2&data_IBS$h2ra_S0=="Yes",],sum)
out7<- as.data.frame(cbind(fru1,fru2))
out7


nas1<-table(data_IBS[data_IBS$Survtime_galst>2&data_IBS$h2ra_S0=="Yes",]$NASIDS,data_IBS[data_IBS$Survtime_galst>2&data_IBS$h2ra_S
0=="Yes",]$galst)
nas2<-aggregate(Survtime_galst~data_IBS[data_IBS$Survtime_galst>2&data_IBS$h2ra_S0=="Yes",]$NASIDS,data_IBS[data_IBS$Survtime_galst
>2&data_IBS$h2ra_S0=="Yes",],sum)
out8<- as.data.frame(cbind(nas1,nas2))
out8


asp1<-table(data_IBS[data_IBS$Survtime_galst>2&data_IBS$h2ra_S0=="Yes",]$ASP,data_IBS[data_IBS$Survtime_galst>2&data_IBS$h2ra_S0==
"Yes",]$galst)
asp2<-aggregate(Survtime_galst~data_IBS[data_IBS$Survtime_galst>2&data_IBS$h2ra_S0=="Yes",]$ASP,data_IBS[data_IBS$Survtime_galst>2&
data_IBS$h2ra_S0=="Yes",],sum)
out9<- as.data.frame(cbind(asp1,asp2))
out9


gerd1<-table(data_IBS[data_IBS$Survtime_galst>2&data_IBS$h2ra_S0=="Yes",]$gerd_b,data_IBS[data_IBS$Survtime_galst>2&data_IBS$h2ra_
S0=="Yes",]$galst)
gerd2<-aggregate(Survtime_galst~data_IBS[data_IBS$Survtime_galst>2&data_IBS$h2ra_S0=="Yes",]$gerd_b,data_IBS[data_IBS$Survtime_gals
t>2&data_IBS$h2ra_S0=="Yes",],sum)
out10<- as.data.frame(cbind(gerd1,gerd2))
out10


pearson_years_ibd<- as.data.frame(cbind(out1, out2,     out3,      out4,      out5,
                                        out6,    out7, out8,      out9,      out10))
pearson_years_ibd

write.table(pearson_years_ibd, file = "D:\\中山七院工作\\文献\\PPI\\cholecystitis\\years_subgroup_h2ra.csv", sep = ",",
```

```
                        col.names = NA,qmethod = "double")



##main analysis--personal years
a1<-table(data_IBS[data_IBS$Survtime_galst>2,]$h2ra_S0,
            data_IBS[data_IBS$Survtime_galst>2,]$galst)
a2<-aggregate(Survtime_galst~data_IBS[data_IBS$Survtime_galst>2,]$h2ra_S0,
                data_IBS[data_IBS$Survtime_galst>2,],sum)
out1<- as.data.frame(cbind(a1,a2))
out1


b1<-table(data_IBS[data_IBS$Survtime_galst>2,]$PPI_S0,
            data_IBS[data_IBS$Survtime_galst>2,]$galst)
b2<-aggregate(Survtime_galst~data_IBS[data_IBS$Survtime_galst>2,]$PPI_S0,
                data_IBS[data_IBS$Survtime_galst>2,],sum)
out2<- as.data.frame(cbind(b1,b2))
out2

pearson_years_ibd<- as.data.frame(cbind(out2, out1))
pearson_years_ibd

write.table(pearson_years_ibd, file = "D:\\中山七院工作\\文献\\PPI\\cholecystitis\\years_main.csv", sep = ",",
                        col.names = NA,qmethod = "double")




####head to head analysis --------

ppi_h<-subset(data_IBS,PPI_H2==2 | PPI_H2==3)
str(ppi_h)
ppi_h$PPI_H2<-ifelse(ppi_h$PPI_H2==3,0,1)
ppi_h$PPI_H2<-factor(ppi_h$PPI_H2,levels = c(0,1),labels = c( "only H2","only PPI" ))


p_h1<-table(ppi_h[ppi_h$Survtime_galst>2,]$PPI_H2,
              ppi_h[ppi_h$Survtime_galst>2,]$galst)
p_h2<-aggregate(Survtime_galst~ppi_h[ppi_h$Survtime_galst>2,]$PPI_H2,
                  ppi_h[ppi_h$Survtime_galst>2,],sum)
head<- as.data.frame(cbind(p_h1,p_h2))
head

write.table(head, file = "D:\\中山七院工作\\文献\\PPI\\cholecystitis\\head_years.csv", sep = ",",
                        col.names = NA,qmethod = "double")


fit<- coxph(Surv(Survtime_galst,galst)~ PPI_H2
```

```
                  +strata(agecat)+strata(gender)+strata(centre)
                  +eth +IDM+bmic
                  +smoking+drinking4+Phy_act+fruit_g
                  +HBP+chol_h+diabete_b+index
                  +ASP+NASIDS+statin+ACEI+ARBs+beteb+vitamin+mineral+Health_R+illness_L
                  ,data=ppi_h)
HR <- round(exp(coef(fit)), 2)
CI <- round(exp(confint(fit)), 2)
P <- round(coef(summary(fit))[,5], 3)
colnames(CI) <- c("Lower", "Higher")
out<- as.data.frame(cbind(HR, CI, P))
out_head2<- out[1,]
out_head2


#sensitive analysis--lag for 4 years--------

a1<-table(data_IBS[data_IBS$Survtime_galst>4,]$PPI_S0,
          data_IBS[data_IBS$Survtime_galst>4,]$galst)
a2<-aggregate(Survtime_galst~data_IBS[data_IBS$Survtime_galst>4,]$PPI_S0,
              data_IBS[data_IBS$Survtime_galst>4,],sum)
out1<- as.data.frame(cbind(a1,a2))
out1


b1<-table(data_IBS[data_IBS$Survtime_galst>4,]$h2ra_S0,
          data_IBS[data_IBS$Survtime_galst>4,]$galst)
b2<-aggregate(Survtime_galst~data_IBS[data_IBS$Survtime_galst>4,]$h2ra_S0,
              data_IBS[data_IBS$Survtime_galst>4,],sum)
out2<- as.data.frame(cbind(b1,b2))
out2


pearson_years_ibd<- as.data.frame(cbind(out1, out2))
pearson_years_ibd

write.table(pearson_years_ibd, file = "D:\\中山七院工作\\文献\\PPI\\cholecystitis\\years_lag4.csv", sep = ",",
                  col.names = NA,qmethod = "double")



fit<- coxph(Surv(Survtime_galst,galst)~PPI_S0
                  +strata(agecat)+strata(gender)+strata(centre)
                  +eth +IDM+bmic
                  +smoking+drinking4+Phy_act+fruit_g
                  +HBP+chol_h+diabete_b+index
                  +ASP+NASIDS+statin+ACEI+ARBs+beteb+vitamin+mineral+Health_R+illness_L
                  ,data=data_IBS[data_IBS$Survtime_galst>4,])
HR <- round(exp(coef(fit)), 2)
```

```
CI <- round(exp(confint(fit)), 2)
P <- round(coef(summary(fit))[,5], 3)
colnames(CI) <- c("Lower", "Higher")
out<- as.data.frame(cbind(HR, CI, P))
out1<- out[1,]
out1



fit<- coxph(Surv(Survtime_galst,galst)~h2ra_S0
            +strata(agecat)+strata(gender)+strata(centre)
            +eth +IDM+bmic
            +smoking+drinking4+Phy_act+fruit_g
            +HBP+chol_h+diabete_b+index
            +ASP+NASIDS+statin+ACEI+ARBs+beteb+vitamin+mineral+Health_R+illness_L
            ,data=data_IBS[data_IBS$Survtime_galst>4,])
HR <- round(exp(coef(fit)), 2)
CI <- round(exp(confint(fit)), 2)
P <- round(coef(summary(fit))[,5], 3)
colnames(CI) <- c("Lower", "Higher")
out<- as.data.frame(cbind(HR, CI, P))
out2<- out[1,]
out2

out_IBS<- as.data.frame(rbind(out1,out2))
out_IBS
write.table(out_IBS, file = "D:\\中山七院工作\\文献\\PPI\\cholecystitis\\lag4.csv", sep = ",",
            col.names = NA,qmethod = "double")









####head to head analysis ceshi--------

ppi_h<-subset(data_rs,PPI_H2==2 | PPI_H2==3)
str(ppi_h)
ppi_h$PPI_H2<-ifelse(ppi_h$PPI_H2==3,0,1)
ppi_h$PPI_H2<-factor(ppi_h$PPI_H2,levels = c(0,1),labels = c( "only H2","only PPI" ))


p_h1<-table(ppi_h[ppi_h$Survtime_galst>2,]$PPI_H2,
            ppi_h[ppi_h$Survtime_galst>2,]$galst)
p_h2<-aggregate(Survtime_galst~ppi_h[ppi_h$Survtime_galst>2,]$PPI_H2,
```

```
                    ppi_h[ppi_h$Survtime_galst>2,],sum)
head1<- as.data.frame(cbind(p_h1,p_h2))
head1



p_h1<-table(ppi_h[ppi_h$Survtime_galst>2&ppi_h$GRS3_c=="Q1",]$PPI_H2,
               ppi_h[ppi_h$Survtime_galst>2&ppi_h$GRS3_c=="Q1",]$galst)
p_h2<-aggregate(Survtime_galst~ppi_h[ppi_h$Survtime_galst>2&ppi_h$GRS3_c=="Q1",]$PPI_H2,
                    ppi_h[ppi_h$Survtime_galst>2&ppi_h$GRS3_c=="Q1",],sum)
head2<- as.data.frame(cbind(p_h1,p_h2))
head2



p_h1<-table(ppi_h[ppi_h$Survtime_galst>2&ppi_h$GRS3_c=="Q2",]$PPI_H2,
               ppi_h[ppi_h$Survtime_galst>2&ppi_h$GRS3_c=="Q2",]$galst)
p_h2<-aggregate(Survtime_galst~ppi_h[ppi_h$Survtime_galst>2&ppi_h$GRS3_c=="Q2",]$PPI_H2,
                    ppi_h[ppi_h$Survtime_galst>2&ppi_h$GRS3_c=="Q2",],sum)
head3<- as.data.frame(cbind(p_h1,p_h2))
head3


p_h1<-table(ppi_h[ppi_h$Survtime_galst>2&ppi_h$GRS3_c=="Q3",]$PPI_H2,
               ppi_h[ppi_h$Survtime_galst>2&ppi_h$GRS3_c=="Q3",]$galst)
p_h2<-aggregate(Survtime_galst~ppi_h[ppi_h$Survtime_galst>2&ppi_h$GRS3_c=="Q3",]$PPI_H2,
                    ppi_h[ppi_h$Survtime_galst>2&ppi_h$GRS3_c=="Q3",],sum)
head4<- as.data.frame(cbind(p_h1,p_h2))
head4


pearson_years_ibd<- as.data.frame(cbind(head1, head2,head3,head4))
pearson_years_ibd

write.table(pearson_years_ibd, file = "D:\\中山七院工作\\文献\\PPI\\cholecystitis\\years_head.csv", sep = ",",
               col.names = NA,qmethod = "double")



fit<- coxph(Surv(Survtime_galst,galst)~ PPI_H2
               +strata(agecat)+strata(gender)+strata(centre)
               +eth +IDM+bmic
               +smoking+drinking4+Phy_act+fruit_g
               +HBP+chol_h+diabete_b
               +ASP+NASIDS+statin+ACEI+ARBs+beteb+vitamin+mineral+Health_R+illness_L
               ,data=ppi_h)
HR <- round(exp(coef(fit)), 2)
CI <- round(exp(confint(fit)), 2)
P <- round(coef(summary(fit))[,5], 3)
```

```
colnames(CI) <- c("Lower", "Higher")
out<- as.data.frame(cbind(HR, CI, P))
out_head0<- out[1,]
out_head0


fit<- coxph(Surv(Survtime_galst,galst)~ PPI_H2
            +strata(agecat)+strata(gender)+strata(centre)
            +eth +IDM+bmic
            +smoking+drinking4+Phy_act+fruit_g
            +HBP+chol_h+diabete_b
            +ASP+NASIDS+statin+ACEI+ARBs+beteb+vitamin+mineral+Health_R+illness_L
            ,data=ppi_h[ppi_h$GRS3_c=="Q1",])
HR <- round(exp(coef(fit)), 2)
CI <- round(exp(confint(fit)), 2)
P <- round(coef(summary(fit))[,5], 3)
colnames(CI) <- c("Lower", "Higher")
out<- as.data.frame(cbind(HR, CI, P))
out_head1<- out[1,]
out_head1

fit<- coxph(Surv(Survtime_galst,galst)~ PPI_H2
            +strata(agecat)+strata(gender)+strata(centre)
            +eth +IDM+bmic
            +smoking+drinking4+Phy_act+fruit_g
            +HBP+chol_h+diabete_b
            +ASP+NASIDS+statin+ACEI+ARBs+beteb+vitamin+mineral+Health_R+illness_L
            ,data=ppi_h[ppi_h$GRS3_c=="Q2",])
HR <- round(exp(coef(fit)), 2)
CI <- round(exp(confint(fit)), 2)
P <- round(coef(summary(fit))[,5], 3)
colnames(CI) <- c("Lower", "Higher")
out<- as.data.frame(cbind(HR, CI, P))
out_head2<- out[1,]
out_head2


fit<- coxph(Surv(Survtime_galst,galst)~ PPI_H2
            +strata(agecat)+strata(gender)+strata(centre)
            +eth +IDM+bmic
            +smoking+drinking4+Phy_act+fruit_g
            +HBP+chol_h+diabete_b
            +ASP+NASIDS+statin+ACEI+ARBs+beteb+vitamin+mineral+Health_R+illness_L
            ,data=ppi_h[ppi_h$GRS3_c=="Q3",])
HR <- round(exp(coef(fit)), 2)
CI <- round(exp(confint(fit)), 2)
P <- round(coef(summary(fit))[,5], 3)
colnames(CI) <- c("Lower", "Higher")
out<- as.data.frame(cbind(HR, CI, P))
```

```
out_head3<- out[1,]
out_head3


out_IBS<- as.data.frame(rbind(out_head0,out_head1,out_head2,out_head3))
out_IBS
write.table(out_IBS, file = "D:\\中山七院工作\\文献\\PPI\\cholecystitis\\head_head.csv", sep = ",",
            col.names = NA,qmethod = "double")



fit<- coxph(Surv(age1,Survtime_galst+age1,galst)~PPI_S0
            +rs1260326+rs11887534+rs4245791+rs9843304_C+rs6471717_G+rs2547231
            +strata(agecat)+strata(gender)+strata(centre)+strata(gerd_b)
            +eth +IDM+bmic
            +smoking+drinking4+Phy_act+fruit_g
            +HBP+chol_h+diabete_b+index
            +Health_R+illness_L
            +ASP+NASIDS+statin+ACEI+ARBs+beteb+vitamin+mineral
            ,data=data_rs[data_rs$Survtime_galst>2,])
HR <- round(exp(coef(fit)), 2)
CI <- round(exp(confint(fit)), 2)
P <- round(coef(summary(fit))[,5], 3)
colnames(CI) <- c("Lower", "Higher")
out<- as.data.frame(cbind(HR, CI, P))
out




####应该用 logit 回归函数，保持与基因风险模型一致
fit<-glm(formula = galst~ age1+gen+strata(centre)
         +eth +IDM+BMI
         +smk+drk+act+fruit_g
         +HBP+chol_h+diabete_b+index
         +heal+ill
         +ASP+NASIDS+statin+ACEI+ARBs+beteb+vitamin+mineral,family = binomial(),
         data=data_rs[data_rs$Survtime_galst>2,])
summary(fit)



data_rs$ECS<-data_rs$age1*(0.0258421)+data_rs$gen*(-0.4173157)+
  data_rs$white*(0.3765274)+data_rs$BMI*0.0651760+
  data_rs$smk*0.0710948+data_rs$drk*(-0.1469631)+data_rs$act*(-0.0964)+
  data_rs$ind*0.4750741+data_rs$heal*(-0.2292650)+
  data_rs$ill*(0.1611549)
summary(data_rs$ECS)
```

```
data_rs$ECS_c<-eqcut(data_rs$ECS,ngroups = 4) ##按三分位数分层

table(data_rs$ECS_c)

data_rs$ECS_c<-factor(data_rs$ECS_c,levels = c("1st quartile of data_rs$ECS: [0.807,2.34)" ,
                                                      "2nd quartile of data_rs$ECS: [2.34,2.70)" ,
                                                      "3rd quartile of data_rs$ECS: [2.70,3.13)",
                                                      "4th quartile of data_rs$ECS: [3.13,6.55]"),
                      labels=c("Q1", "Q2","Q3","Q4"))
table(data_rs$ECS_c)


#####For PPI
fit<- coxph(Surv(age1,Survtime_galst+age1,galst)~PPI_S0
            +strata(agecat)+strata(gender)+strata(centre)
            +eth +IDM+bmic
            +smoking+drinking4+Phy_act+fruit_g
            +HBP+chol_h+diabete_b+index
            +Health_R+illness_L
            +ASP+NASIDS+statin+ACEI+ARBs+beteb+vitamin+mineral
            ,data=data_rs[data_rs$Survtime_galst>2&data_rs$ECS_c=="Q1",])
HR <- round(exp(coef(fit)), 2)
CI <- round(exp(confint(fit)), 2)
P <- round(coef(summary(fit))[,5], 3)
colnames(CI) <- c("Lower", "Higher")
out<- as.data.frame(cbind(HR, CI, P))
out1<- out[1,]
out1

fit<- coxph(Surv(age1,Survtime_galst+age1,galst)~PPI_S0
            +strata(agecat)+strata(gender)+strata(centre)
            +eth +IDM+bmic
            +smoking+drinking4+Phy_act+fruit_g
            +HBP+chol_h+diabete_b+index
            +Health_R+illness_L
            +ASP+NASIDS+statin+ACEI+ARBs+beteb+vitamin+mineral
            ,data=data_rs[data_rs$Survtime_galst>2&data_rs$ECS_c=="Q2",])
HR <- round(exp(coef(fit)), 2)
CI <- round(exp(confint(fit)), 2)
P <- round(coef(summary(fit))[,5], 3)
colnames(CI) <- c("Lower", "Higher")
out<- as.data.frame(cbind(HR, CI, P))
out2<- out[1,]
out2


fit<- coxph(Surv(age1,Survtime_galst+age1,galst)~PPI_S0
            +strata(agecat)+strata(gender)+strata(centre)
            +eth +IDM+bmic
```

```
              +smoking+drinking4+Phy_act+fruit_g
              +HBP+chol_h+diabete_b+index
              +Health_R+illness_L
              +ASP+NASIDS+statin+ACEI+ARBs+beteb+vitamin+mineral
              ,data=data_rs[data_rs$Survtime_galst>2&data_rs$ECS_c=="Q3",])
HR <- round(exp(coef(fit)), 2)
CI <- round(exp(confint(fit)), 2)
P <- round(coef(summary(fit))[,5], 3)
colnames(CI) <- c("Lower", "Higher")
out<- as.data.frame(cbind(HR, CI, P))
out3<- out[1,]
out3


fit<- coxph(Surv(age1,Survtime_galst+age1,galst)~PPI_S0
              +strata(agecat)+strata(gender)+strata(centre)+strata(gerd_b)
              +eth +IDM+bmic
              +smoking+drinking4+Phy_act+fruit_g
              +HBP+chol_h+diabete_b+index
              +Health_R+illness_L
              +ASP+NASIDS+statin+ACEI+ARBs+beteb+vitamin+mineral
              ,data=data_rs[data_rs$Survtime_galst>2&data_rs$ECS_c=="Q4",])
HR <- round(exp(coef(fit)), 2)
CI <- round(exp(confint(fit)), 2)
P <- round(coef(summary(fit))[,5], 3)
colnames(CI) <- c("Lower", "Higher")
out<- as.data.frame(cbind(HR, CI, P))
out4<- out[1,]
out4

out_IBS<- as.data.frame(rbind(out1,out2,out3,out4))
out_IBS


write.table(out_IBS, file = "D:\\中山七院工作\\文献\\PPI\\cholecystitis\\ECS_ppi.csv", sep = ",",
              col.names = NA,qmethod = "double")


#####For H2RA
fit<- coxph(Surv(age1,Survtime_galst+age1,galst)~h2ra_S0
              +strata(agecat)+strata(gender)+strata(centre)+strata(gerd_b)
              +eth +IDM+bmic
              +smoking+drinking4+Phy_act+fruit_g
              +HBP+chol_h+diabete_b+index
```

```
                  +Health_R+illness_L
                  +ASP+NASIDS+statin+ACEI+ARBs+beteb+vitamin+mineral
                  ,data=data_rs[data_rs$Survtime_galst>2&data_rs$ECS_c=="Q1",])
HR <- round(exp(coef(fit)), 2)
CI <- round(exp(confint(fit)), 2)
P <- round(coef(summary(fit))[,5], 3)
colnames(CI) <- c("Lower", "Higher")
out<- as.data.frame(cbind(HR, CI, P))
out1<- out[1,]
out1

fit<- coxph(Surv(age1,Survtime_galst+age1,galst)~h2ra_S0
                  +strata(agecat)+strata(gender)+strata(centre)+strata(gerd_b)
                  +eth +IDM+bmic
                  +smoking+drinking4+Phy_act+fruit_g
                  +HBP+chol_h+diabete_b+index
                  +Health_R+illness_L
                  +ASP+NASIDS+statin+ACEI+ARBs+beteb+vitamin+mineral
                  ,data=data_rs[data_rs$Survtime_galst>2&data_rs$ECS_c=="Q2",])
HR <- round(exp(coef(fit)), 2)
CI <- round(exp(confint(fit)), 2)
P <- round(coef(summary(fit))[,5], 3)
colnames(CI) <- c("Lower", "Higher")
out<- as.data.frame(cbind(HR, CI, P))
out2<- out[1,]
out2


fit<- coxph(Surv(age1,Survtime_galst+age1,galst)~h2ra_S0
                  +strata(agecat)+strata(gender)+strata(centre)+strata(gerd_b)
                  +eth +IDM+bmic
                  +smoking+drinking4+Phy_act+fruit_g
                  +HBP+chol_h+diabete_b+index
                  +Health_R+illness_L
                  +ASP+NASIDS+statin+ACEI+ARBs+beteb+vitamin+mineral
                  ,data=data_rs[data_rs$Survtime_galst>2&data_rs$ECS_c=="Q3",])
HR <- round(exp(coef(fit)), 2)
CI <- round(exp(confint(fit)), 2)
P <- round(coef(summary(fit))[,5], 3)
colnames(CI) <- c("Lower", "Higher")
out<- as.data.frame(cbind(HR, CI, P))
out3<- out[1,]
out3


fit<- coxph(Surv(age1,Survtime_galst+age1,galst)~h2ra_S0
                  +strata(agecat)+strata(gender)+strata(centre)+strata(gerd_b)
                  +eth +IDM+bmic
                  +smoking+drinking4+Phy_act+fruit_g
```

```
                +HBP+chol_h+diabete_b+index
                +Health_R+illness_L
                +ASP+NASIDS+statin+ACEI+ARBs+beteb+vitamin+mineral
                ,data=data_rs[data_rs$Survtime_galst>2&data_rs$ECS_c=="Q4",])
HR <- round(exp(coef(fit)), 2)
CI <- round(exp(confint(fit)), 2)
P <- round(coef(summary(fit))[,5], 3)
colnames(CI) <- c("Lower", "Higher")
out<- as.data.frame(cbind(HR, CI, P))
out4<- out[1,]
out4

out_IBS<- as.data.frame(rbind(out1,out2,out3,out4))
out_IBS


write.table(out_IBS, file = "D:\\中山七院工作\\文献\\PPI\\cholecystitis\\ECS_h2ra.csv", sep = ",",
                col.names = NA,qmethod = "double")






## PS score------
fit<- coxph(Surv(age1,Survtime_galst+age1,galst)~PPI_S0
                +strata(agecat)+strata(gender)+strata(centre)
                +eth +IDM+bmic
                +smoking+drinking4+Phy_act+fruit_g
                +HBP+chol_h+diabete_b+index
                +Health_R+illness_L
                +ASP+NASIDS+statin+ACEI+ARBs+beteb+vitamin+mineral
                ,data=data_IBS[data_IBS$Survtime_galst>2,])



library(MatchIt)
vars2<-c("n_eid", "PPI_S0","h2ra_S0","age1","gender" ,"IDM","bmic","eth","centre",
        "smoking","drinking4","Phy_act","fruit_g","index",
        "HBP","chol_h","diabete_b","Health_R","illness_L",
        "ASP","NASIDS","vitamin","mineral","Survtime_galst","galst","agecat",
        "statin","ACEI","ARBs","beteb"
)
data_PS<-data_IBS[vars2]
summary(data_PS)
data_PS<-data_PS[is.na(data_PS$IDM)=="FALSE",]       #######必须要删除空缺值
summary(data_PS)
```

```
summary(is.na(data_PS$IDM))
set.seed(2020)

log1<-glm(formula = PPI_S0~agecat+gender+strata(centre)
          +eth +IDM+bmic
          +smoking+drinking4+Phy_act+fruit_g
          +HBP+chol_h+diabete_b+index
          +Health_R+illness_L
          +ASP+NASIDS+statin+ACEI+ARBs+beteb+vitamin+mineral,family = binomial(),
          data=data_PS)
PS<-fitted(log1)
SD(logit(PS))
0.2*SD(logit(PS))    ###这个即为卡钳值 caliper



psmmatch1=matchit(PPI_S0~agecat+gender+strata(centre)
                  +eth +IDM+bmic
                  +smoking+drinking4+Phy_act+fruit_g
                  +HBP+chol_h+diabete_b+index
                  +Health_R+illness_L
                  +ASP+NASIDS+statin+ACEI+ARBs+beteb+vitamin+mineral, method="nearest",ratio=4,caliper=0.2706794,
                  data=data_PS)    ####进行 PS 匹配

summary(psmmatch1)   ###可以计算 PS 匹配后每个变量的标准化差异

matchdata1=match.data(psmmatch1) ##得到匹配之后的数据集（可用于后续分析）
summary(matchdata1)
fit<- coxph(Surv(age1,age1+Survtime_galst,galst)~PPI_S0
            ,data=matchdata1[matchdata1$Survtime_galst>2,])
HR <- round(exp(coef(fit)), 2)
CI <- round(exp(confint(fit)), 2)
P <- round(coef(summary(fit))[,5], 3)
colnames(CI) <- c("Lower", "Higher")
out<- as.data.frame(cbind(HR, CI, P))
out1<- out[1,]
out1




###PS for h2ra_S0
log2<-glm(formula = h2ra_S0~agecat+gender+strata(centre)
          +eth +IDM+bmic
          +smoking+drinking4+Phy_act+fruit_g
          +HBP+chol_h+diabete_b+index
          +Health_R+illness_L
          +ASP+NASIDS+statin+ACEI+ARBs+beteb+vitamin+mineral,family = binomial(),
          data=data_PS)
PS2<-fitted(log2)
```

```
SD(logit(PS2))
0.2*SD(logit(PS2))      ###这个即为卡钳值  caliper



psmmatch2=matchit(h2ra_S0~agecat+gender+strata(centre)
                              +eth +IDM+bmic
                              +smoking+drinking4+Phy_act+fruit_g
                              +HBP+chol_h+diabete_b+index
                              +Health_R+illness_L
                              +ASP+NASIDS+statin+ACEI+ARBs+beteb+vitamin+mineral, method="nearest",ratio=4,caliper=0.2706794,
                              data=data_PS)      ####进行 PS 匹配

summary(psmmatch2)    ###可以计算 PS 匹配后每个变量的标准化差异

matchdata2=match.data(psmmatch2) ##得到匹配之后的数据集（可用于后续分析）
summary(matchdata2)

fit<- coxph(Surv(age1,age1+Survtime_galst,galst)~h2ra_S0
                    ,data=matchdata2[matchdata2$Survtime_galst>2,])
HR <- round(exp(coef(fit)), 2)
CI <- round(exp(confint(fit)), 2)
P <- round(coef(summary(fit))[,5], 3)
colnames(CI) <- c("Lower", "Higher")
out<- as.data.frame(cbind(HR, CI, P))
out2<- out[1,]
out2

table(matchdata1[matchdata1$Survtime_galst>2,]$PPI_S0,matchdata1[matchdata1$Survtime_galst>2,]$galst)
aggregate(Survtime_galst~matchdata1[matchdata1$Survtime_galst>2,]$PPI_S0,matchdata1[matchdata1$Survtime_galst>2,],sum)

table(matchdata2[matchdata2$Survtime_galst>2,]$h2ra_S0,matchdata2[matchdata2$Survtime_galst>2,]$galst)
aggregate(Survtime_galst~matchdata2[matchdata2$Survtime_galst>2,]$PPI_S0,matchdata2[matchdata2$Survtime_galst>2,],sum)



###########further adjust for ppi genes-----------
gwas_med<-read.csv("D:\\GWAS\\GWAS medication use UK\\gwas 结果\\gwas_med.csv",header=TRUE, sep=",")
vars3<-c("n_eid", "rs1619179_A","rs6965956_G","rs11171710_G","rs3858461_T" ,"rs2051815_G")
gene_ppi<-gwas_med[vars3]
data_PPI<-merge(data_IBS,gene_ppi,by="n_eid")

fit<-glm(formula = PPI_S0~ age1+gen+rs1619179_A+rs6965956_G+rs11171710_G+rs3858461_T+rs2051815_G,family = binomial(),
               data=data_PPI)
summary(fit)



fit<- coxph(Surv(age1,Survtime_galst+age1,galst)~PPI_S0 +rs1619179_A+rs6965956_G+rs11171710_G+rs3858461_T+rs2051815_G
                    +strata(agecat)+strata(gender)+strata(centre)
                    +eth +IDM+bmic
```

```
                    +smoking+drinking4+Phy_act+fruit_g
                    +HBP+chol_h+diabete_b+index
                    +Health_R+illness_L
                    +ASP+NASIDS+statin+ACEI+ARBs+beteb+vitamin+mineral
                    ,data=data_PPI[data_PPI$Survtime_galst>2,])
HR <- round(exp(coef(fit)), 2)
CI <- round(exp(confint(fit)), 2)
P <- round(coef(summary(fit))[,5], 3)
colnames(CI) <- c("Lower", "Higher")
out<- as.data.frame(cbind(HR, CI, P))
out3<- out[1,]
out3



fit<- coxph(Surv(age1,Survtime_diabete+age1,diabete)~PPI_S0 +rs1619179_A+rs6965956_G+rs11171710_G+rs3858461_T+rs2051815_G
                    +strata(agecat)+strata(gender)+strata(centre)
                    +eth +IDM+bmic
                    +smoking+drinking4+Phy_act+fruit_g
                    +HBP+chol_h+index
                    +Health_R+illness_L
                    +ASP+NASIDS+statin+ACEI+ARBs+beteb+vitamin+mineral
                    ,data=data_PPI[data_PPI$Survtime_diabete>2,])
HR <- round(exp(coef(fit)), 2)
CI <- round(exp(confint(fit)), 2)
P <- round(coef(summary(fit))[,5], 3)
colnames(CI) <- c("Lower", "Higher")
out<- as.data.frame(cbind(HR, CI, P))
out3<- out[1,]
out3


fit<- coxph(Surv(age1,Survtime_IBD+age1,IBD)~PPI_S0 +rs1619179_A+rs6965956_G+rs11171710_G+rs3858461_T+rs2051815_G
                    +strata(agecat)+strata(gender)+strata(centre)
                    +eth +IDM+bmic
                    +smoking+drinking4+Phy_act+fruit_g
                    +HBP+chol_h+index
                    +Health_R+illness_L
                    +ASP+NASIDS+statin+ACEI+ARBs+beteb+vitamin+mineral
                    ,data=data_PPI[data_PPI$Survtime_IBD>2,])
HR <- round(exp(coef(fit)), 2)
CI <- round(exp(confint(fit)), 2)
P <- round(coef(summary(fit))[,5], 3)
colnames(CI) <- c("Lower", "Higher")
out<- as.data.frame(cbind(HR, CI, P))
out3<- out[1,]
out3

######测试----
```

```
fit<- coxph(Surv(age1,Survtime_galst+age1,galst)~PPI_S0
            +strata(agecat)+strata(gender)+strata(centre)
            +eth +IDM+bmic
            +smoking+drinking4+Phy_act+fruit_g
            +HBP+chol_h+diabete_b+index
            +Health_R+illness_L
            +ASP+NASIDS+statin+ACEI+ARBs+beteb+vitamin+mineral
            ,data=data_IBS[data_IBS$Survtime_galst>2&(data_IBS$Health_R=="Excellent"|data_IBS$Health_R=="Good"),])
HR <- round(exp(coef(fit)), 2)
CI <- round(exp(confint(fit)), 2)
P <- round(coef(summary(fit))[,5], 3)
colnames(CI) <- c("Lower", "Higher")
out<- as.data.frame(cbind(HR, CI, P))
out1<- out[1,]
out1


fit<- coxph(Surv(age1,Survtime_galst+age1,galst)~PPI_S0
            +strata(agecat)+strata(gender)+strata(centre)
            +eth +IDM+bmic
            +smoking+drinking4+Phy_act+fruit_g
            +HBP+chol_h+diabete_b+index
            +Health_R+illness_L
            +ASP+NASIDS+statin+ACEI+ARBs+beteb+vitamin+mineral
            ,data=data_IBS[data_IBS$Survtime_galst>2&(data_IBS$Health_R=="Fair"),])
HR <- round(exp(coef(fit)), 2)
CI <- round(exp(confint(fit)), 2)
P <- round(coef(summary(fit))[,5], 3)
colnames(CI) <- c("Lower", "Higher")
out<- as.data.frame(cbind(HR, CI, P))
out2<- out[1,]
out2

fit<- coxph(Surv(age1,Survtime_galst+age1,galst)~PPI_S0
            +strata(agecat)+strata(gender)+strata(centre)
            +eth +IDM+bmic
            +smoking+drinking4+Phy_act+fruit_g
            +HBP+chol_h+diabete_b+index
            +Health_R+illness_L
            +ASP+NASIDS+statin+ACEI+ARBs+beteb+vitamin+mineral
            ,data=data_IBS[data_IBS$Survtime_galst>2&(data_IBS$Health_R=="Poor"),])
HR <- round(exp(coef(fit)), 2)
CI <- round(exp(confint(fit)), 2)
P <- round(coef(summary(fit))[,5], 3)
colnames(CI) <- c("Lower", "Higher")
out<- as.data.frame(cbind(HR, CI, P))
out3<- out[1,]
out3
```

```
fit<- coxph(Surv(age1,Survtime_galst+age1,galst)~PPI_S0
            +strata(agecat)+strata(gender)+strata(centre)
            +eth +IDM+bmic
            +smoking+drinking4+Phy_act+fruit_g
            +HBP+chol_h+diabete_b+index
            +Health_R+illness_L
            +ASP+NASIDS+statin+ACEI+ARBs+beteb+vitamin+mineral
            ,data=data_IBS[data_IBS$Survtime_galst>2&(data_IBS$Health_R=="Poor"|data_IBS$Health_R=="Fair"),])
HR <- round(exp(coef(fit)), 2)
CI <- round(exp(confint(fit)), 2)
P <- round(coef(summary(fit))[,5], 3)
colnames(CI) <- c("Lower", "Higher")
out<- as.data.frame(cbind(HR, CI, P))
out4<- out[1,]
out4
```
